# (Un)common space in infant neuroimaging studies: A systematic review of infant templates

**DOI:** 10.1002/hbm.25816

**Published:** 2022-03-09

**Authors:** Alexander J. Dufford, C. Alice Hahn, Hannah Peterson, Silvia Gini, Saloni Mehta, Alexis Alfano, Dustin Scheinost

**Affiliations:** ^1^ Department of Radiology and Biomedical Imaging Yale School of Medicine New Haven Connecticut USA; ^2^ Department of Psychology Quinnipiac University Hamden Connecticut USA; ^3^ Department of Statistics and Data Science Yale University New Haven Connecticut USA; ^4^ Interdepartmental Neuroscience Program Yale University New Haven Connecticut USA; ^5^ Department of Biomedical Engineering Yale University New Haven Connecticut USA; ^6^ Child Study Center Yale School of Medicine New Haven Connecticut USA

**Keywords:** common space, functional, magnetic resonance imaging, neonatal

## Abstract

In neuroimaging, spatial normalization is an important step that maps an individual's brain onto a template brain permitting downstream statistical analyses. Yet, in infant neuroimaging, there remain several technical challenges that have prevented the establishment of a standardized template for spatial normalization. Thus, many different approaches are used in the literature. To quantify the popularity and variability of these approaches in infant neuroimaging studies, we performed a systematic review of infant magnetic resonance imaging (MRI) studies from 2000 to 2020. Here, we present results from 834 studies meeting inclusion criteria. Studies were classified into (a) processing data in single subject space, (b) using an off the shelf, or “off the shelf,” template, (c) creating a study specific template, or (d) using a hybrid of these methods. We found that across the studies in the systematic review, single subject space was the most used (no common space). This was the most used common space for diffusion‐weighted imaging and structural MRI studies while functional MRI studies preferred off the shelf atlases. We found a pattern such that more recently published studies are more commonly using off the shelf atlases. When considering special populations, preterm studies most used single subject space while, when no special populations were being analyzed, an off the shelf template was most common. The most used off the shelf templates were the UNC Infant Atlases (24%). Using a systematic review of infant neuroimaging studies, we highlight a lack of an established “standard” template brain in these studies.

## Graphical abstract

Spatial normalization to a common space is a critical preprocessing step. However, there is no consensus for common space when imaging infant populations. We provide an overview of the current state of the field in regard to common spaces used.

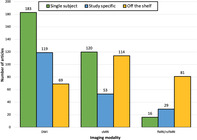

## 1 INTRODUCTION

A critical preprocessing step for the analysis of magnetic resonance imaging (MRI) data is spatial normalization (Friston et al., 1995; Poldrack, Mumford, & Nichols, 2011). Spatial normalization is the process of bringing brain volumes that have been acquired in different individuals into a common neuroanatomical common (or reference) space (Crivello et al., 2002; Fox, Perlmutter, & Raichle, 1985; Poldrack et al., 2011) and is typically performed in analyses across all modalities: structural MRI (Ashburner & Friston, 2000), diffusion MRI (Jones et al., 2002), and functional MRI (Poldrack et al., 2011). Spatial normalization to a common space is often necessary for image statistics to be computed across participants (Friston, 1994; Gee, Alsop, & Aguirre, 1997), which assumes that across participants brain structures occupy the same standard anatomical space in a consistent manner (Fox, 1995; Toga & Thompson, 2001). Spatial normalization is highly dependent on the common space template used and results may not be directly comparable if different templates are used. (Laird et al., 2010; Lancaster et al., 2007; Rohlfing, Sullivan, & Pfefferbaum, 2009). With the goal of rigor and reproducibility in mind, it is crucial for common spaces to be standardized across fields of neuroimaging to compare across studies (Fox, 1995; Friston et al., 1995). For adult neuroimaging studies, two common spaces (along with a standard coordinate system, or stereotaxic space) have emerged as standard common spaces for spatial normalization: Talairach space (Talairach & Tournoux, 1988) and MNI space (Collins, Neelin, Peters, & Evans, 1994; Evans et al., 1993), with MNI space now considered the “standard” (Laird et al., 2010).

In recent years, MRI has had increased utilization as a methodological tool to examine brain development in infancy (Eyre et al., 2020; Gilmore, Knickmeyer, & Gao, 2018; Howell et al., 2019; Li et al., 2019). However, there is no standard common space template for infant brain studies. The adult MNI atlas is not appropriate for infant studies primarily due to vast neuroanatomical differences between the adult and infant brain. (Gaillard, Grandin, & Xu, 2001) Studies have shown the use of the adult MNI atlas in the analysis of infant neuroimaging studies introduces significant biases (Kazemi, Moghaddam, Grebe, Gondry‐Jouet, & Wallois, 2007). There also exists major challenges in the development of a standardized infant brain common space. For example, brain development during the first year of life is rapid and dynamic with specific anatomical patterns of development for different ages (Gilmore et al., 2007; Knickmeyer et al., 2008). Second, high quality neuroimaging data is difficult to acquire in infancy due to low spatial resolution, low tissue contrast, and high participant motion (Shi et al., 2011; Xue et al., 2007). Third, common space templates are typically constructed based upon a large sample of high‐quality neuroimaging data, thus making a template difficult to construct in infants (Shi et al., 2011). With these existing challenges, approaches to spatial normalization for infant neuroimaging studies have been largely inconsistent (Li et al., 2019; Oishi, Chang, & Huang, 2019; Shi et al., 2011). While “standard” infant atlases have been proposed (Oishi et al., 2019; Shi et al., 2011), it is currently unclear which common space approaches are used most frequently in infant neuroimaging. In this systematic review, we conducted a comprehensive literature search, review of common spaces used by each study, and analysis of common spaces for infant neuroimaging studies published between the years 2000–2020. By conducting this systematic review, we sought to understand the current state of the infant neuroimaging field in terms of the popularity and variability in spatial normalization methodology and to assist in the field of infant neuroimaging to adopt a “standard” common space moving forward.

## 2 METHODS

### 2.1 Objective

In this systematic review, we aimed to summarize the approaches to spatial normalization used in infant neuroimaging studies between the years 2000 and 2020. We only included original quantitative research studies in the systematic review.

### 2.2 Eligibility criteria

Quantitative research studies were excluded from the systematic review if they were: (a) published before the year 2000 (b) written in languages other than English (c) animal studies, case reports, review articles, clinical/radiologist review, not MRI of the brain, and methodological manuscripts (d) fetal MRI studies or participants were older than 18 months chronological age (e) articles using only other imaging modalities other than MRI, (e.g., fNIRS, PET, EEG).

### 2.3 Search procedure and studies identified

**FIGURE 1:**
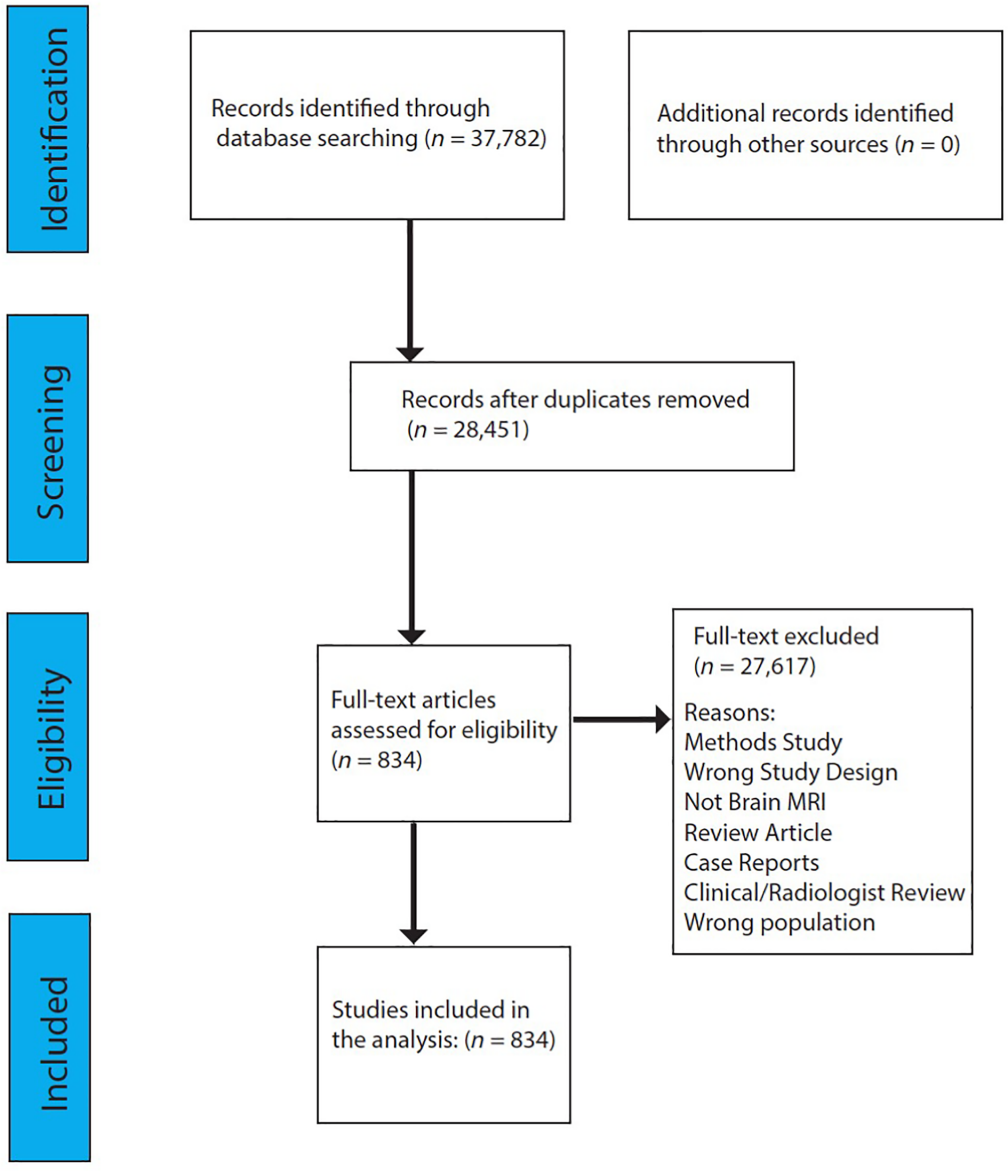
PRISMA diagram for the systematic review of infant common spaces

We conducted a search on PubMed for infant neuroimaging studies that fit the eligibility criteria. Literature was compiled on September 2–3rd, 2020 using the following search string: “infant MRI” and “neonatal MRI”, “neonatal ‘fmri’”, “toddler ‘fmri’”, “‘toddler fmri’”, “preterm fmri”, “neonate(s)”, “infant(s)”, “(((infant) OR (neonate)) Or (newborn)) AND ((fmri) OR (MRI) OR (DTI))”. The initial search resulted in 37,782 manuscripts. The authors conducted screening and eligibility assessment based upon the eligibility criteria previously described using the web‐tool Rayyan (Ouzzani, Hammady, Fedorowicz, & Elmagarmid, 2016). After the screening procedure had identified a subset of manuscripts that fit the eligibility criteria (834), the full articles were reviewed for eligibility and coded into four categories based upon the common space utilized in the study: (a) single subject space (e.g., analyses conducted in native space or no common space was used), (b) a study specific common space such that the common space was generated using the data in the study (e.g., tract‐based spatial statistics or TBSS option “‐n”), (c) an “off the shelf” atlas was used as the common space (e.g., the UNC Neonate Atlas), or (d) a hybrid approach to common space was utilized (e.g., more than one common space within the same imaging modality or different common spaces were used for each imaging modality). The results from the screening procedure are shown in the PRISMA Consort Chart (see Figure 1). We examined the breakdowns of common space by imaging modality: diffusion‐weighted imaging (DWI), structural MRI, or functional MRI (fMRI)/resting‐state (rsfMRI). Further, we examined the distributions of imaging modalities by publication year, common space by publication year, common space by age at scan, and common space by special population. For the studies that utilized off the shelf atlases, we examined the breakdown of which atlases were most used. All authors contributed to the rating these articles.

### 2.4 Statistical methods

For the analyses of common space (overall), by imaging modality, by age of the sample, by population, and by year, the distribution of “off the shelf” atlases, we used the count for each generated from the review of the full article. For a subset of the articles (*n* = 298, articles from 2018–2020), we calculated the inter‐rater reliability (Cohen's kappa) of the agreement of two raters for classify articles into a common space category. For the calculation of Cohen's kappa, we used the Kappa() function in the R package “irr” (Gamer, Lemon, Gamer, Robinson, & Kendall's, 2012). To test for differences in distributions for two‐way contingency tables, we used a chi‐square test. To test for differences in distributions for three‐way contingency tables, such as common space (single subject, study specific, off the shelf) by imaging modality (DWI, sMRI, and fMRI) by publication year (before 2011 or 2011 and after), the log‐linear analysis version of a chi‐square test was used. The log‐linear analysis version of the chi‐square test is recommended for three‐way contingency tables and calculates *G* ^2^ via a likelihood‐ratio chi‐square. *G* ^2^ is like *X* ^2^ but is based on the ratio of the observed to the expected frequencies. For a three‐way contingency table of rows (A), columns (B), and layers (C), seven *G* ^2^ values can be calculated. ABC calculates the significance of a three‐way interaction of A, B, and C. The two‐way interactions are each calculated (AB, AC, BC) and three values are calculated for the two‐way interactions with the effect of the third removed such that AB(C) is the two‐way interaction of AB removing the effect of C. We reported the interactions of BC if they are significant, but do not interpret these interactions as we are only focused on the interactions of common space. *p*‐values were corrected for multiple comparisons using Bonferroni correction.

## 3 RESULTS

### 3.1 Inter‐rater reliability

The analysis of the inter‐rater reliability indicated “very good” agreement between the two raters: *K* = 0.945, *p* <.0001.

### 3.2 Common space by imaging modality

**FIGURE 2:**
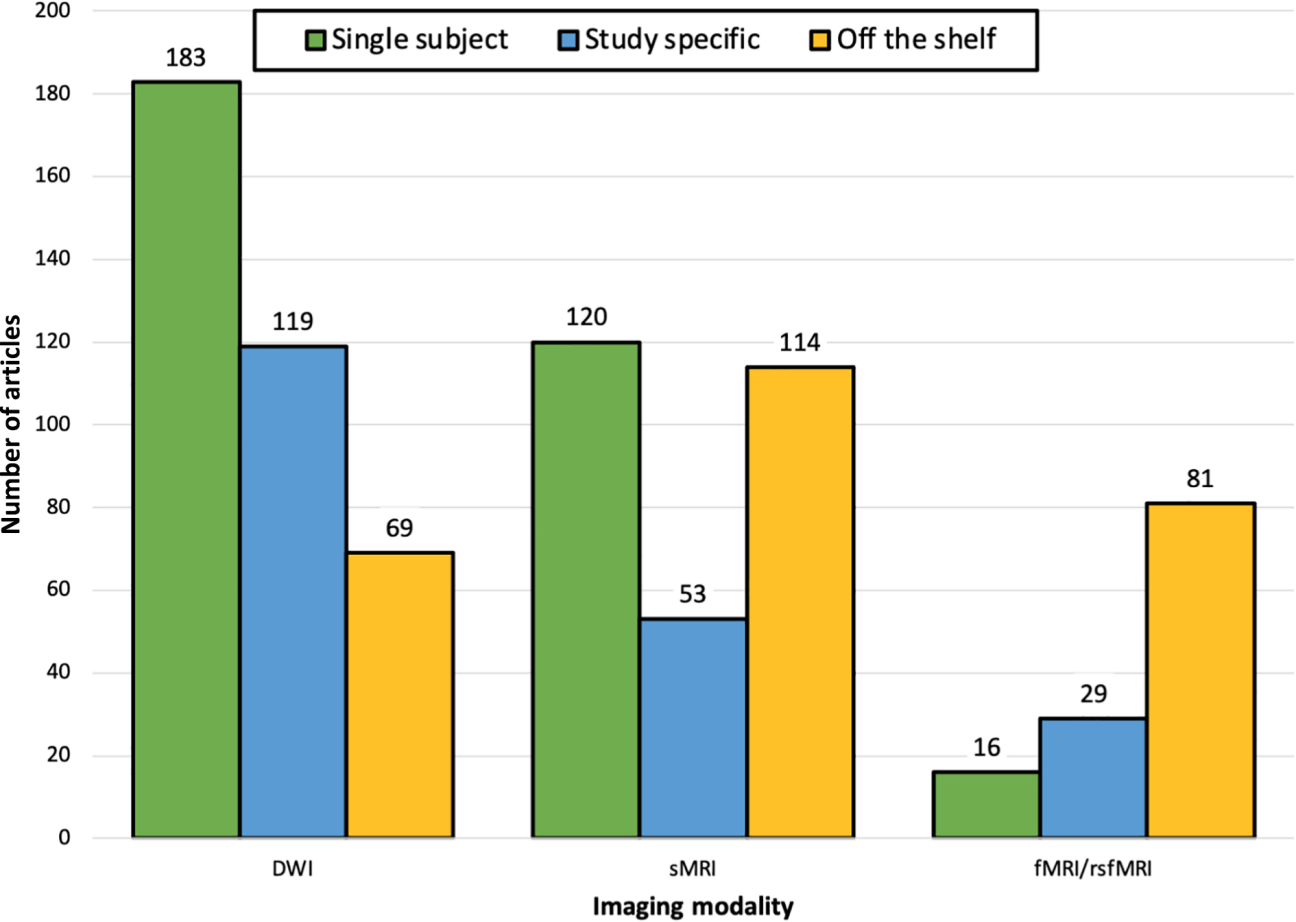
Frequency distribution of different common space registrations grouped by imaging modality and across all modalities (Total). The DWI category includes studies employing diffusion tensor imaging (DTI), diffusion kurtosis imaging (DKI), and any other modality based upon DWI (e.g., neurite orientation dispersion and density imaging). The sMRI category refers to structural MRI studies, and fMRI/rsfMRI to task and resting state functional MRI studies. A single study can be counted twice if multiple modalities were used

According to our systematic review, the most used common space across imaging modalities was single subject space, followed by off the shelf atlases, study specific templates, and lastly by hybrid registrations (see Figure 2). We examined the differences in distributions for common space (single subject, study specific, and off the shelf) by imaging modality (DWI, sMRI, fMRI) using a 3 × 3 contingency table. For the contingency table analyses, we removed any studies that used a “hybrid” approach to common space (*n* = 50), this resulted in 784 studies for this analysis. The log‐linear test for the 3 × 3 contingency table was significant (*X* ^2^[4, 784] = 106.46, *p* <.0001), indicating that the different modalities relied on different common space approaches. Upon further inspection, in DWI studies single subject space appeared to be the most popular (49.32% of all DWI studies), followed by study specific (32%), and off the shelf (18%). For structural MRI studies, single subject space and off the shelf atlases represented the majority (with 41.8% and 39.72% prevalence, respectively), followed by registration to a study specific template (18.46%). For fMRI and rsfMRI studies, off the shelf templates were the most common approach (64.2%), followed by study specific (23%). Analyses in single subject space (12.6%) were the least common for this imaging modality. Results of the common space by imaging modality analysis suggest that, across the studies included in the systematic review, the different modalities used different common spaces, most studies used single subject space (40.6%), and DWI was the most common imaging modality (47.32%).

### 3.3 Common space by imaging modality by publication year

**FIGURE 3:**
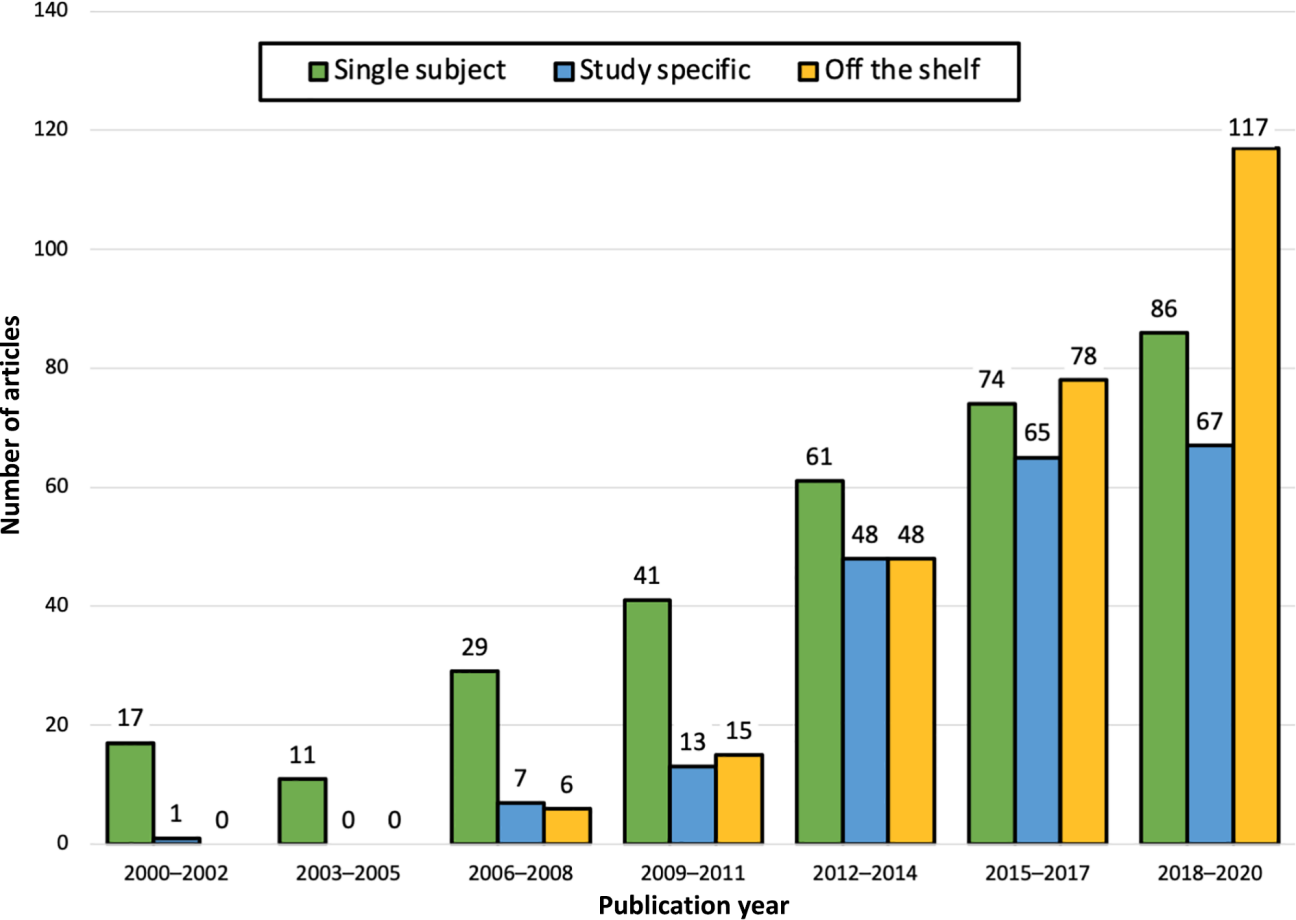
Frequency of imaging modalities across 20 years of infant MRI publications ranging from years 2000 to 2020. Distribution was analyzed in 3‐year increments

**FIGURE 4:**
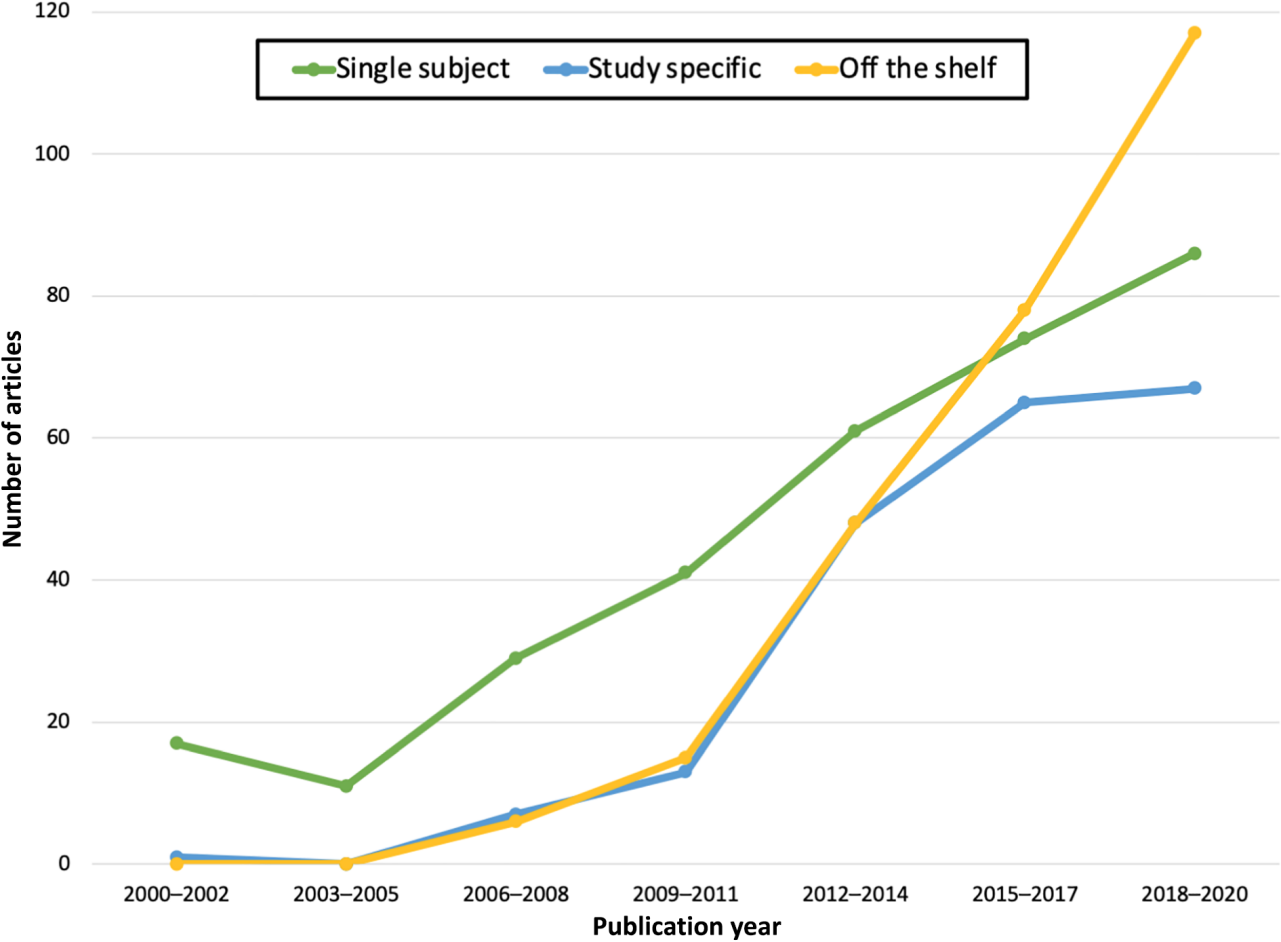
Studies published prior to 2010 primarily used single subject space compared to using a template—either off the shelf or study specific (single subject space: *n* = 83, study specific: *n* = 18, off the shelf: *n* = 12). In contrast, studies published after 2011 primarily used a template (single subject space: *n* = 236, study specific: *n* = 183, off the shelf template: *n* = 252)

To study trends over time in the field for common space and imaging modality, studies were coded based upon their year of publication. To examine potential common space by imaging modality by publication year interactions we binarized publication year to studies between 2000–2010 and 2011–2020. Overall, there were significantly more infant MRI studies published from 2011 to 2020 than the decade before. Using a likelihood‐ratio chi‐square, we examined the interactions of common space, imaging modality, and publication year using a 3 (common space: single subject, study specific, off the shelf) by 3 (imaging modality: DWI, sMRI, fMRI) by 2 (publication year: 2000–2010 and 2011–2020) contingency table. The *G*‐test indicated a significant common space by imaging modality by publication year interaction (*G* ^2^[12, 784] = 187.3, *p* <.0001). Further, while controlling the effect of the third variable, there was a significant common space by imaging modality interaction (*G* ^2^[8, 784] = 121.94, *p* <.0001) and common space by publication year interaction (*G* ^2^[6, 784] = 71.5, *p* <.0001) but not a significant imaging modality by publication year interaction (*G* ^2^[6, 784] = 12.76, *p* = 0.05). Overall, these interactions suggest that, while the relative popularity of the imaging modalities did not change across these two time points (Figure 3), what common spaces that were used did changed, with off the shelf atlases becoming the most popular choice by 2020 (Figure 4).

### 3.4 Common space by imaging modality by age at scan

**FIGURE 5:**
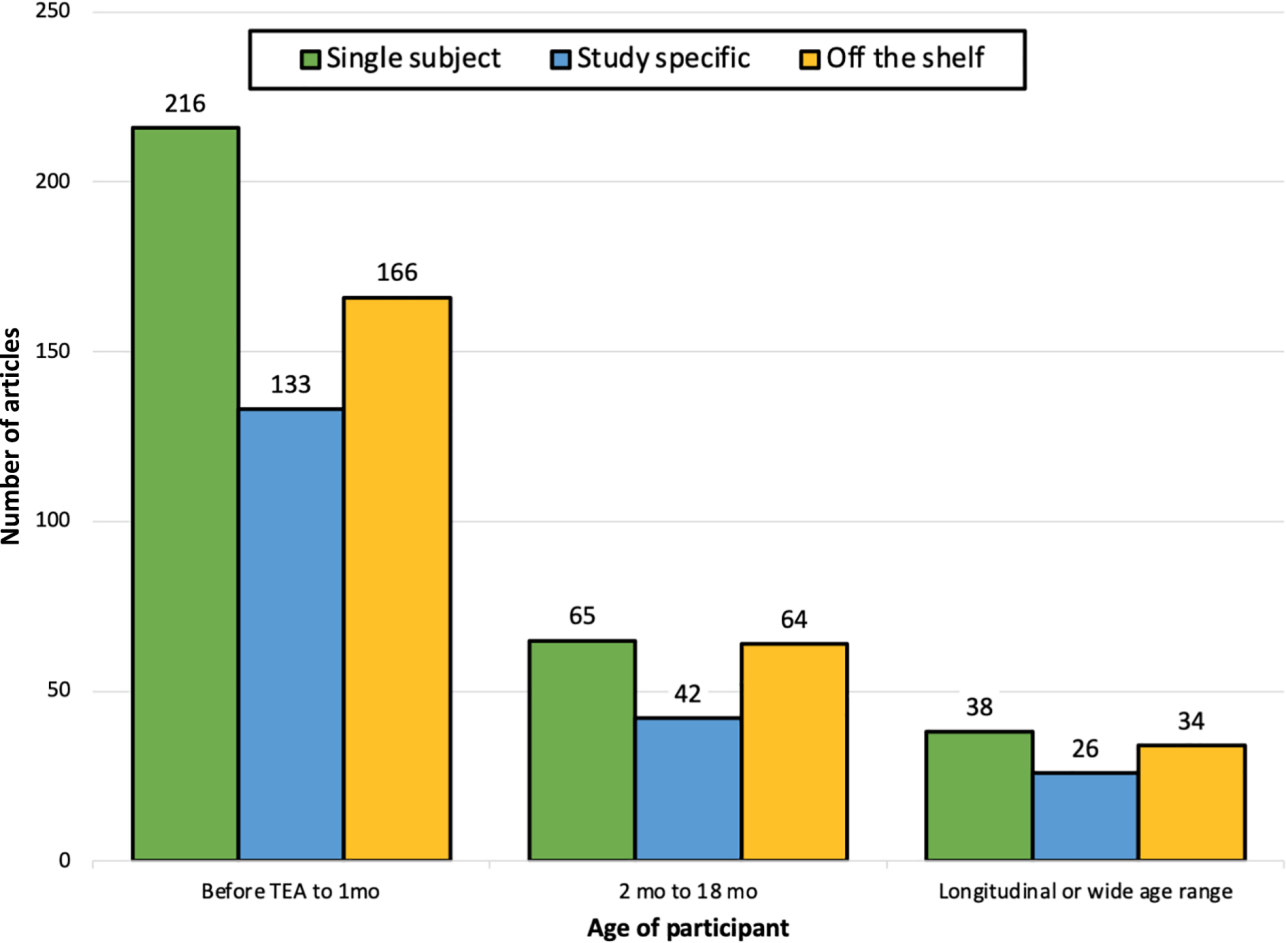
Frequency distribution of common space registrations across age groups. The distribution was analyzed by grouping studies in which infants were scanned before term equivalent age (TEA) to 1 month old and studies in which infants were scanned at 2–18 months of age. Longitudinal scans and studies utilizing a wide age range were accounted for separately

For this analysis, we classified the studies into age groups based upon the age at scan. Before term‐equivalent age (TEA) referred to studies in which the sample was less than 37 weeks postmenstrual age (PMA) at the time of scan. Two additional categories were examined which included “longitudinal scans” and “wide age range.” Longitudinal scans refer to studies that involve the same population being imaged at multiple time points. The wide age range category consisted of samples with age ranges greater than 5 months or cross‐sectional data collected at multiple timepoints. Studies were coded as a longitudinal or wide age range studies, studies with infants that were before TEA–1 month old at the age of scan, and studies with infants that were 2–18 months at the time of the scan. We examined the three‐way interaction of these variables using a 3 (common space: single subject, study specific, off the shelf) by 3 (imaging modality: DWI, sMRI, fMRI) by 3 (age at scan: longitudinal or wide age range, before TEA–1 month, or 2–18‐month‐old). The *G*‐test indicated a significant common space by imaging modality by age at scan interaction (*G* ^2^[20, 784] = 198.06, *p* <.0001). Controlling for the effect of the third variable, there was a significant common space by imaging modality interaction (*G* ^2^[12, 784] = 115.54, *p* <.0001), a significant common space by age at scan interaction (*G* ^2^[12, 784] = 66.76, *p* <.0001), and a significant imaging modality by age at scan interaction (*G* ^2^[12, 784] = 50.32, *p* <.0001. The testing of these interactions suggests different common spaces were used at different age ranges with studies of older infants (>2 months of age at the time of the scan) and longitudinal studies using off the shelf templates more commonly that studies of younger infants (<2 months of age at the time of scan; Figure 5).

### 3.5 Common space by imaging modality by special population

**FIGURE 6:**
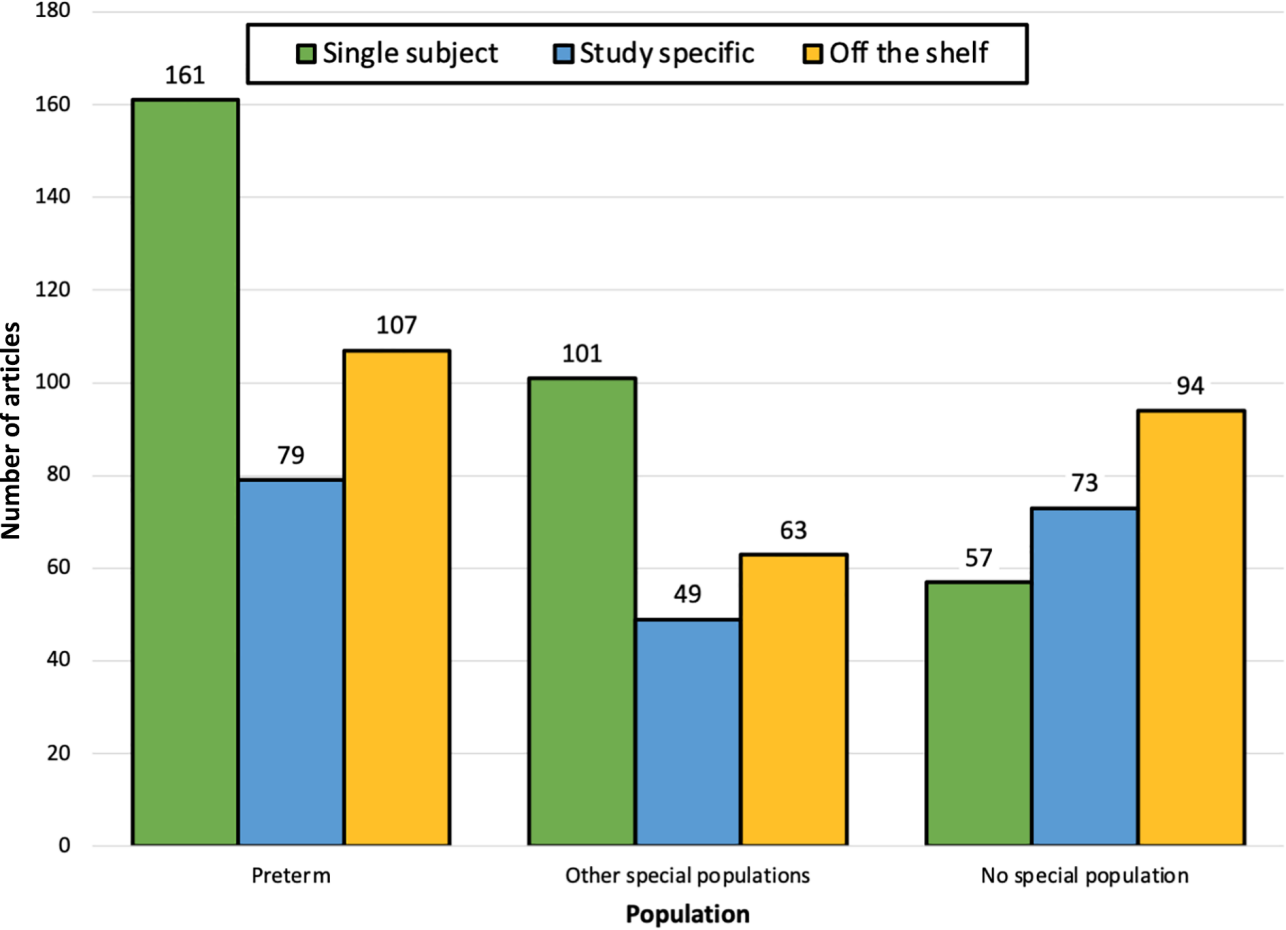
Frequency distribution (Panel A) and percentage breakdown (Panel B) of type of common space registration across types of population in the sample. Samples were classified as “preterm” if they included infants who were less than 37 weeks gestational age at birth and low birth weight infants, “other special populations” if they included infants with neurodevelopmental disorders, medical conditions, or prenatal exposures, or “no special population” if they only included typically developing infants

As infant studies may choose a specific type of common space for certain special populations of interest, we determined the number of studies using each common space for studies of preterm infants, other special populations besides preterm, and studies that did not have a special population (see Figure 6). The preterm category was determined by if the sample of infants imaged was less than 37 weeks gestational age at birth and included studies of low‐birth‐weight infants. The other special populations category included infants with autism spectrum disorder (ASD)/high risk for ASD, hypoxic–ischemic encephalopathy, prenatal exposure to alcohol/drugs/maternal mood symptoms, intrauterine growth restriction, and congenital heart diseases. These clinical categories did not have enough studies for individual group analysis. We tested the common space by imaging modality by special population interaction using a 3 (common space: single subject, study specific, off the shelf) by 3 (imaging modality: DWI, sMRI, fMRI) by 3 (special population: preterm, other special population, no special population). The *G*‐test indicated a significant common space by imaging modality by age at scan interaction (*G* ^2^[20, 784] = 176.36, *p* <.0001). Controlling for the effect of the third variable, there was a significant common space by imaging modality interaction (*G* ^2^[12, 784] = 109.42, *p* <.0001), a significant common space by special population interaction (*G* ^2^[12, 784] = 28.44, *p* <.004), and significant imaging modality by special population interaction (*G* ^2^[12, 784] = 32.3, *p* <.001). Overall, these suggest that the common space choice significantly differed between studies of with or without special populations. In preterm studies and studies of other special populations, the most popular common space is single subject space. In contrast, the off the shelf common space is most popular when the study is comprised of no special population.

### 3.6 Off the shelf atlas distribution

**FIGURE 7:**
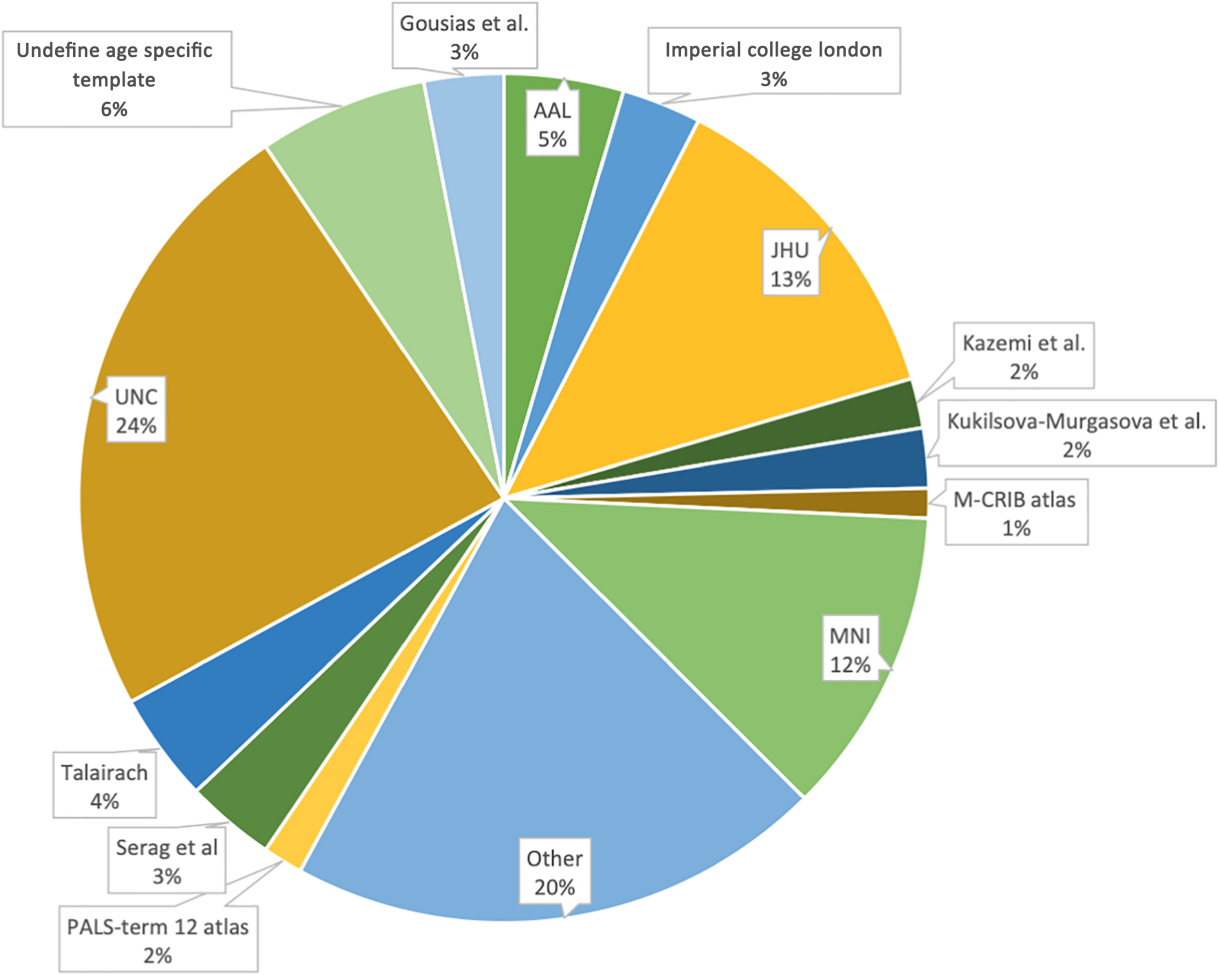
The most common off the shelf templates used were UNC infant atlases (*n* = 62), JHU neonate atlases (*n* = 34) and other; templates that were used infrequently (*n* = 54)

Of the 264 studies classified as using an off the shelf atlas, we examined the breakdown of which atlases were the most used. The most common off the shelf atlas was the UNC infant 0–1–2 atlases (Shi et al., 2011; http://www.med.unc.edu/bric/ideagroup/free-softwares/unc-infant-0-1-2-atlases) used in 24% of the studies, followed by 20% of the studies fitting into an “other” category for off the shelf atlases, followed by 13% that used the JHU neonate atlases (Oishi et al., 2011; http://cmrm.med.jhmi.edu/cmrm/Data_neonate_atlas/atlas_neonate.htm; see Figure 7).

## 4 DISCUSSION

In our systematic review of the common spaces used for infant neuroimaging between years 2000–2020, several patterns emerged. DWI and sMRI studies mostly used single subject space while fMRI studies have mostly used an off the shelf atlas. While early studies relied many on single subject space, off the shelf atlases increased in popularity across the last decade and were the most popular by 2018–2020. We found that single subject space was most common for studies with young infants (before TEA–1 month old) with an increase use of off the shelf atlases in older samples and in longitudinal samples (multiple scan time points across infancy). For the examination of common spaces for special populations, studies of preterm infants and other special populations favored single subject space while studies without a special population most used an off the shelf template. For studies that used an off the shelf atlas, the UNC infant atlases were the most common, followed by the JHU neonate atlases. The findings of the systematic review indicate a need for a standardization across studies regarding a common space, that will be critical for rigor and reproducibility as the field of infant neuroimaging matures.

There still exist several challenges specific to infant neuroimaging data that have contributed to the lack of consensus and standardization of a common space across the field (Korom et al., 2021). A standard common space for infant neuroimaging would have to consider the rapid growth in cytoarchitecture, shape, and volume that occurs between birth and the end of the second year of life (Oishi et al., 2019). Further, compared to adult data, infant neuroimaging data has reduced tissue contrast between gray and white matter (Gilmore et al., 2018). The relative intensities of gray and white matter for T1‐ and T2‐weighted images are similar between 4 and 8 months of age, posing issues for analyzes that involve tissue classification (Gilmore et al., 2018). In addition to poor image quality and tissue contrast, standardization of a common space for infant neuroimaging would require both a T1‐weighted and T2‐weighted template as researchers may only collect (or prioritize) a T1 or T2 structural image based upon the age of the sample. Lastly, the common space would have to be representative and consist of many high‐quality scans. For an off the shelf common space atlases, greater than 100 images averaged is typically required (Shi et al., 2011). If this was conducted for multiple age ranges, likely, >100 high quality images acquired longitudinally would be needed. This is exceptionally difficult when imaging infants. However, as infant neuroimaging datasets increase in size, such as the Baby Connectome Project and Developing Human Connectome Project, a standard common space will be critical.

A standard common space for infant neuroimaging would need to address the existing limitations discussed above. It would also be useful for this common space to have correspondence to the adult MNI coordinate system. Most neuroimaging results are based upon adult studies (mostly registered to the adult MNI template; Oishi et al., 2019). Therefore, having these as a reference with direct anatomical correspondence can enhance our understanding of the developing infant brain. Our results indicated a shift in the field moving from single subject space analysis to using off the shelf atlases. This finding is encouraging as both single subject space and study specific common spaces may be highly biased by the sample (S. Zhang & Arfanakis, 2013). Due to the replicability crisis (Gorgolewski & Poldrack, 2016; Klapwijk, van den Bos, Tamnes, Raschle, & Mills, 2021), it is critical for the field of infant neuroimaging to reduce as much sample‐specific bias as possible to enhance rigor and reproducibility across studies. A standard common space for registration with a standard coordinate system for reporting results would facilitate meta‐analyses and data sharing. For example, to directly compare the results of two studies, a research lab may need to re‐register their data into the common space another lab used. This is not only time consuming but can introduce bias. Similarly, coordinate‐based meta‐analyses—which provide a more precise estimate of the effect size and can increase the generalizability of the results of individual studies—are not possible without a common coordinate system to report results. As infant neuroimaging has an existing small sample size issue (Korom et al., 2021), defining a standard common space will facilitate meta‐analyses and data sharing and enhance rigor and reproducibility across infant neuroimaging studies.

When defining this standard common space, it is natural to ask: are currently off the shelf atlas sufficient or do new ones need to be created? The UNC infant 0–1–2 (Shi et al., 2011) and JHU neonate atlases (Oishi et al., 2011) are the most used. The UNC infant atlases are examples of spatio‐temporal atlases that have a neonatal, 1 year old and 2‐year‐old atlas. The atlases were generated from 95 neonates that were scanned 5 weeks after birth and then scanned at 1 and 2 years old. The atlases are available in both T1‐weighted and T2‐weighted images, tissue probability maps, and an infant Automated Anatomical Labeling (AAL) parcellation (Oishi et al., 2019; Shi et al., 2011; Tzourio‐Mazoyer et al., 2002) The collection of JHU neonate atlases includes a group averaged atlas and single‐subject‐based atlas for T1‐ and T2‐weighted images as well as a DTI based atlas. The group averaged atlases for the DTI and T2‐weighted atlas were constructed from 20 healthy, term‐birth, neonates scanned within 4 days after birth. The JHU neonate T1 atlas was constructed from 15 healthy, term‐birth, neonates (37–41 gestational weeks). In terms of frequency of use, adopting the UNC infant atlases as the standard of the field could allow for future studies to be comparable to the greatest of the studies conducted to date in terms of common space. The spatio‐temporal aspect of the UNC atlases is also appealing as three major developmental periods in early life are represented: neonatal, 1 year old, and 2 years old. However, the UNC infant atlases (0–1–2) are limited if the age of the study's participants are outside these three time points (e.g., 5–6‐months‐old, 17–18‐months‐old). To mitigate this issue, spatiotemporal longitudinal 0–3–6–9–12 months‐old atlases as both T1‐ and T2‐weighted images have been released (https://www.nitrc.org/projects/infant_atlas_4d/; Zhang et al., 2016). Nevertheless, given the need for large sample sizes and fine grain age‐specificity, these atlases may just be the starting points in developing a standard common space.

Along with the standardization of a common space (or spaces in the case of spatio‐temporal atlases), the growing field of infant neuroimaging will have to adopt a standardized method of choosing an off the shelf template. For example, if two studies both have a sample of infants with a mean chronological age of 6 months and one study chooses a neonate atlas as its common space and the other chooses a 1‐year atlas, the results may not be directly comparable despite the ages of the infants being similar. The choice of the different atlas for common space registration could negatively impact the rigor and reproducibility of the studies as the normalization procedure could provide differences between atlases in noise due to misregistration of the images at the voxel level and impact statistical power (Oishi et al., 2019). Standardization, not only the common space, but also how to best account for participant ages will be critical.

Infant neuroimaging datasets begin to reach “big data” levels like adult neuroimaging data. Two large open‐source infant neuroimaging datasets exist: the Baby Connectome Project (Howell et al., 2019) and the Developing Human Connectome Project (Eyre et al., 2020). Further, the National Institutes of Health (NIH) has announced the HEALthy Brain and Child Development Study (HBCD). The HBCD will involve recruitment of a large, diverse sample of pregnant women across several sites in the United States. It will include neuroimaging data with a focus on characterizing developmental trajectories. Both within and between these large infant neuroimaging datasets consensus on common space registration (for multiple ages) will be critical to combine these valuable data.

Studies in the field of infant neuroimaging have steadily increased since the beginning of use for nonclinical studies in the early 1990s (an average of 160 publications per year to 530 per year in the last decade) (Pollatou et al., 2022). However, due to the challenges of infant neuroimaging, the field has experienced a lag in standardization of best practices compared to adult MRI studies. As we have demonstrated with the current systematic review, there is a critical need for the field to establish a standard common space. To address these issues concerning establishing best practices for the field, organizations, like *Fetal*, *Infant*, *Toddler*, *Neuroimaging Group* (FIT'NG) (Pollatou et al., under review), will be critical to establish best practices within the field (common space, scan time, prep procedures for scanning), community exchange and collaboration (sharing analytic pipelines, datasets), and education (training across institutions at multiple levels).

The current systematic review is not without its limitations. Some of the literature currently under review lacked a clear description of methodology, thus making it hard to identify the type of registration, age range, and population. This was especially true for studies developing a study specific template. In addition, many studies used multiple common spaces or adapted an off the shelf template for their own use that was later labeled as “hybrid” or “other.” This definition did not account for the use of different common spaces for unique modalities within the same paper. Furthermore, there are some methodological limitations within our review, such as limited data on inter‐rater reliability. Inclusion and exclusion inter‐rater reliability were 100% when calculated in a subset of 370 papers rated by two reviewers. Clear exclusion and inclusion criteria provided guidelines and limited discrepancies between raters reviewing papers, mitigating any inter‐rater reliability issues, but future reviews might benefit from collecting information about reviewer's agreement. Finally, given the large number of papers included in our analyses, a small number of mis‐classified papers is unlikely to change the general trends reported here.

## 5 CONCLUSIONS

Despite these limitations, our systematic review provides evidence of a lack of a standard common space for infant neuroimaging studies. With the maturation of the field of infant neuroimaging, a standard common space will be critical to examine the generalizability of results across samples, ages, special populations, and imaging modalities. Further, a standard common space has the potential to increase rigor and reproducibility by reducing sample specific bias. The results of the systematic review have provided a quantification of the last two decades of infant neuroimaging to gauge where the field currently stands in terms of common space. With the results of the review in mind and an eye toward the future of the field including large consortium neuroimaging datasets, we suggest it is a critical time to adopt a standard common space.

## AUTHOR CONTRIBUTIONS

Dustin Scheinost conceptualized the study. Alexander J. Dufford, C. Alice Hahn, Hannah Peterson, Silvia Gini, Saloni Mehta, Alexis Alfano, and Dustin Scheinost analyzed the data. Alexander J. Dufford, C. Alice Hahn, Hannah Peterson, and Dustin Scheinost wrote the manuscript.

## Supporting information


**Appendix** S1: Supporting Information

## Data Availability

The *R* script used for the analysis of the systematic review data is available at: (https://github.com/ajdneuro12/CommonSpace). The list of articles included in the analysis is available in Supporting Information.
