# Supplementary material for: (Un)common space in infant neuroimaging studies: A systematic review of infant templates
Source: Hum Brain Mapp. 2022 Mar 9;43(9):3007–16. doi: 10.1002/hbm.25816 (PMC9120551; doi:10.1002/hbm.25816)
Supplement: Supplementary file 1 — Appendix S1: Supporting Information [file HBM-43-3007-s001.docx]

**Supporting Information**

1. Difference in the metabolic response to photic stimulation of the lateral geniculate nucleus and the primary visual cortex of infants: a fMRI study.
2. A milestone for normal development of the infantile brain detected by functional MRI.
3. Quantitative comparison of intrabrain diffusion in adults and preterm and term neonates and infants.
4. Neonatal hypoxic-ischemic encephalopathy: detection with diffusion-weighted MR imaging.
5. Neonatal auditory activation detected by functional magnetic resonance imaging.
6. Microstructural brain development after perinatal cerebral white matter injury assessed by diffusion tensor magnetic resonance imaging.
7. Impaired cerebral cortical gray matter growth after treatment with dexamethasone for neonatal chronic lung disease.
8. The effects of repeated antenatal glucocorticoid therapy on the developing brain.
9. Functional brain imaging using fMRI and optical topography in infancy.
10. Serial quantitative diffusion tensor MRI of the premature brain: development in newborns with and without injury.
11. Age-dependent change in metabolic response to photic stimulation of the primary visual cortex in infants: functional magnetic resonance imaging study.
12. Prediction of adverse outcome with cerebral lactate level and apparent diffusion coefficient in infants with perinatal asphyxia.
13. Diffusion-tensor MR imaging of gray and white matter development during normal human brain maturation.
14. Study of pediatric brain development using magnetic resonance imaging of anisotropic diffusion.
15. Radial organization of developing preterm human cerebral cortex revealed by non-invasive water diffusion anisotropy MRI.
16. Evaluation of normal age-related changes in anisotropy during infancy and childhood as shown by diffusion tensor imaging.
17. Changes in brain water diffusion during the 1st year of life.
18. Functional neuroimaging of speech perception in infants.
19. Isotropic apparent diffusion coefficient mapping of postnatal cerebral development.
20. T2 relaxation values in the developing preterm brain.
21. Comparisons of regional white matter diffusion in healthy neonates and adults performed with a 3.0-T head-only MR imaging unit.
22. Diffusion-weighted imaging of the brain in preterm infants with focal and diffuse white matter abnormality.
23. Diffusion-weighted MR imaging in the early diagnosis of periventricular leukomalacia.
24. Early experience alters brain function and structure.
25. Prolonged T*2 values in newborn versus adult brain: Implications for fMRI studies of newborns.
26. 3 Tesla magnetic resonance imaging of the brain in newborns.
27. Diffusion-weighted imaging and proton magnetic resonance spectroscopy in perinatal hypoxic-ischemic encephalopathy: association with neuromotor outcome at 18 months of age.
28. Quantitative diffusion tensor MRI fiber tractography of sensorimotor white matter development in premature infants.
29. Comparing microstructural and macrostructural development of the cerebral cortex in premature newborns: diffusion tensor imaging versus cortical gyration.
30. Somatosensory lateralization in the newborn brain.
31. Assessment of the early organization and maturation of infants' cerebral white matter fiber bundles: a feasibility study using quantitative diffusion tensor imaging and tractography.
32. Detailed semiautomated MRI based morphometry of the neonatal brain: preliminary results.
33. Reduced fractional anisotropy on diffusion tensor magnetic resonance imaging after hypoxic-ischemic encephalopathy.
34. Pediatric diffusion tensor imaging: normal database and observation of the white matter maturation in early childhood.
35. Magnetization transfer ratio in the brain of preterm subjects: age-related changes during the first 2 years of life.
36. MR imaging, MR spectroscopy, and diffusion tensor imaging of sequential studies in neonates with encephalopathy.
37. Early assessment of brain maturation by MR imaging segmentation in neonates and premature infants.
38. Regional brain development in serial magnetic resonance imaging of low-risk preterm infants.
39. Perinatal risk factors altering regional brain structure in the preterm infant.
40. Different neuronal networks are associated with spikes and slow activity in hypsarrhythmia.
41. Displacement of brain regions in preterm infants with non-synostotic dolichocephaly investigated by MRI.
42. The normal neonatal brain: MR imaging, diffusion tensor imaging, and 3D MR spectroscopy in healthy term neonates.
43. Neonatal microstructural development of the internal capsule on diffusion tensor imaging correlates with severity of gait and motor deficits.
44. Fractional anisotropy in white matter tracts of very-low-birth-weight infants.
45. Relationship between white matter apparent diffusion coefficients in preterm infants at term-equivalent age and developmental outcome at 2 years.
46. Early growth in brain volume is preserved in the majority of preterm infants.
47. Serial diffusion tensor imaging detects white matter changes that correlate with motor outcome in premature infants.
48. Diffusion tensor imaging assessment of brain white matter maturation during the first postnatal year.
49. Apparent diffusion coefficient pseudonormalization time in neonatal hypoxic-ischemic encephalopathy.
50. Abnormal brain development in newborns with congenital heart disease.
51. Early postnatal development of corpus callosum and corticospinal white matter assessed with quantitative tractography.
52. Early myelination patterns in the brainstem auditory nuclei and pathway: MRI evaluation study.
53. Regional gray matter growth, sexual dimorphism, and cerebral asymmetry in the neonatal brain.
54. Resting-state networks in the infant brain.
55. Diffusion tensor imaging with tract-based spatial statistics reveals local white matter abnormalities in preterm infants.
56. Intrauterine growth restriction affects the preterm infant's hippocampus.
57. Functional connectivity of the sensorimotor area in naturally sleeping infants.
58. Neonatal probabilistic models for brain, CSF and skull using T1-MRI data: preliminary results.
59. Head growth in preterm infants: correlation with magnetic resonance imaging and neurodevelopmental outcome.
60. Changes of the corpus callosum in children who suffered perinatal injury of the periventricular crossroads of pathways.
61. Preterm infant hippocampal volumes correlate with later working memory deficits.
62. Diffusion tensor imaging of the pyramidal tracts in infants with motor dysfunction.
63. Quantitative DTI assessment of periventricular white matter changes in neonatal meningitis.
64. Prominent signal intensity of T1/T2 prolongation in subcortical white matter of the anterior temporal region on conventional screening MRI of late preterm infants with normal development.
65. Comparative evaluation of the cerebral and cerebellar white matter development in pediatric age group using quantitative diffusion tensor imaging.
66. Diffusion-weighted and conventional MR imaging in neonatal hypoxic ischemia: two-year follow-up study.
67. Early myelination patterns in the central auditory pathway of the higher brain: MRI evaluation study.
68. Primary cortical folding in the human newborn: an early marker of later functional development.
69. Asynchrony of the early maturation of white matter bundles in healthy infants: quantitative landmarks revealed noninvasively by diffusion tensor imaging.
70. A structural MRI study of human brain development from birth to 2 years.
71. Assessment of functional development in normal infant brain using arterial spin labeled perfusion MRI.
72. Probabilistic diffusion tractography of the optic radiations and visual function in preterm infants at term equivalent age.
73. Spontaneous brain activity in the newborn brain during natural sleep--an fMRI study in infants born at full term.
74. Age-related grey matter changes in preterm infants: an MRI study.
75. Cerebellar development in the preterm neonate: effect of supratentorial brain injury.
76. Impaired neuroanatomic development in infants with congenital heart disease.
77. Functional magnetic resonance imaging of the sensorimotor system in preterm infants.
78. Abnormal white matter signal on MR imaging is related to abnormal tissue microstructure.
79. Diffusion tensor imaging detects abnormalities in the corticospinal tracts of neonates with infantile Krabbe disease.
80. Neonatal brain structure on MRI and diffusion tensor imaging, sex, and neurodevelopment in very-low-birthweight preterm children.
81. Fractional anisotropy for assessment of white matter tracts injury in methylmalonic acidemia.
82. Quantitative fiber tracking analysis of the optic radiation correlated with visual performance in premature newborns.
83. MR-determined hippocampal asymmetry in full-term and preterm neonates.
84. Brain development of the preterm neonate after neonatal hydrocortisone treatment for chronic lung disease.
85. Amygdala enlargement in toddlers with autism related to severity of social and communication impairments.
86. Females follow a more "compact" early human brain development model than males. A case-control study of preterm neonates.
87. Interleukin-6 -174 and -572 genotypes and the volume of deep gray matter in preterm infants.
88. Volumetric and anatomical MRI for hypoxic-ischemic encephalopathy: relationship to hypothermia therapy and neurosensory impairments.
89. Structural asymmetries in the infant language and sensori-motor networks.
90. Maturation of thalamic radiations between 34 and 41 weeks' gestation: a combined voxel-based study and probabilistic tractography with diffusion tensor imaging.
91. Evidence on the emergence of the brain's default network from 2-week-old to 2-year-old healthy pediatric subjects.
92. Temporal and spatial development of axonal maturation and myelination of white matter in the developing brain.
93. Mapping growth patterns and genetic influences on early brain development in twins.
94. Longitudinal analysis of neural network development in preterm infants.
95. Language or music, mother or Mozart? Structural and environmental influences on infants' language networks.
96. Spatio-Temporal Analysis of Early Brain Development.
97. Genetic and environmental contributions to neonatal brain structure: A twin study.
98. Diffusion tensor imaging of the cortical plate and subplate in very-low-birth-weight infants.
99. Early sensitivity training for parents of preterm infants: impact on the developing brain.
100. A common neonatal image phenotype predicts adverse neurodevelopmental outcome in children born preterm.
101. Structural asymmetries in motor and language networks in a population of healthy preterm neonates at term equivalent age: a diffusion tensor imaging and probabilistic tractography study.
102. Tractography-based quantitation of corticospinal tract development in premature newborns.
103. Diffusion-weighted imaging of cerebral white matter and the cerebellum following preterm birth.
104. Efficiency of fractional anisotropy and apparent diffusion coefficient on diffusion tensor imaging in prognosis of neonates with hypoxic-ischemic encephalopathy: a methodologic prospective pilot study.
105. Quantitative fiber tracking of the optic radiation is correlated with visual-evoked potential amplitude in preterm infants.
106. Associations between the size of the amygdala in infancy and language abilities during the preschool years in normally developing children.
107. Correlation of apparent diffusion coefficient and fractional anisotropy values in the developing infant brain.
108. White matter changes in extremely preterm infants, a population-based diffusion tensor imaging study.
109. Caffeine and brain development in very preterm infants.
110. Somatosensory cortical activation identified by functional MRI in preterm and term infants.
111. Tract-based spatial statistics of magnetic resonance images to assess disease and treatment effects in perinatal asphyxial encephalopathy.
112. Changes of MR and DTI appearance in early human brain development.
113. Functional specializations for music processing in the human newborn brain.
114. Emergence of resting state networks in the preterm human brain.
115. Twin-singleton differences in neonatal brain structure.
116. Differential effects of intrauterine growth restriction on brain structure and development in preterm infants: a magnetic resonance imaging study.
117. Early specialization for voice and emotion processing in the infant brain.
118. Diffuse periventricular leukomalacia in preterm children: assessment of grey matter changes by MRI.
119. The functional architecture of the infant brain as revealed by resting-state fMRI.
120. Brain anatomical networks in early human brain development.
121. Prematurity affects cortical maturation in early childhood.
122. Assessment of cortical visual impairment in infants with periventricular leukomalacia: a pilot event-related FMRI study.
123. Temporal and spatial evolution of brain network topology during the first two years of life.
124. Morphometric differences in the Heschl's gyrus of hearing impaired and normal hearing infants.
125. Tractography of developing white matter of the internal capsule and corpus callosum in very preterm infants.
126. Characterization of the corpus callosum in very preterm and full-term infants utilizing MRI.
127. Neural language networks at birth.
128. Fiber tracking at term displays gender differences regarding cognitive and motor outcome at 2 years of age in preterm infants.
129. Development of corpus callosum in preterm infants is affected by the prematurity: in vivo assessment of diffusion tensor imaging at term-equivalent age.
130. Biphasic time course of brain water ADC observed during the first month of life in term neonates with severe perinatal asphyxia is indicative of poor outcome at 3 years.
131. MR imaging and outcome of term neonates with perinatal asphyxia: value of diffusion-weighted MR imaging and ¹H MR spectroscopy.
132. Neonatal intensive care unit stress is associated with brain development in preterm infants.
133. Gender differences in language and motor-related fibers in a population of healthy preterm neonates at term-equivalent age: a diffusion tensor and probabilistic tractography study.
134. A robust cerebral asymmetry in the infant brain: the rightward superior temporal sulcus.
135. Neuro-developmental outcome at 18 months in premature infants with diffuse excessive high signal intensity on MR imaging of the brain.
136. Does diffusion tensor imaging-based tractography at 3 months of age contribute to the prediction of motor outcome after perinatal arterial ischemic stroke?
137. Early maturation of the linguistic dorsal pathway in human infants.
138. Perinatal cortical growth and childhood neurocognitive abilities.
139. Development trends of white matter connectivity in the first years of life.
140. Longitudinal regression analysis of spatial-temporal growth patterns of geometrical diffusion measures in early postnatal brain development with diffusion tensor imaging.
141. Diffusion tensor imaging in preterm infants with punctate white matter lesions.
142. Population differences in brain morphology and microstructure among Chinese, Malay, and Indian neonates.
143. The effect of preterm birth on thalamic and cortical development.
144. White matter heritability using diffusion tensor imaging in neonatal brains.
145. Tract-based spatial statistics (TBSS): application to detecting white matter tract variation in mild hypoxic-ischemic neonates.
146. Development of BOLD signal hemodynamic responses in the human brain.
147. Neurologic outcomes in very preterm infants undergoing surgery.
148. Deep gray matter maturation in very preterm neonates: regional variations and pathology-related age-dependent changes in magnetization transfer ratio.
149. Longitudinal development of cortical and subcortical gray matter from birth to 2 years.
150. Comparison of cerebral volume in children aged 18-22 and 36-47 months born preterm and term.
151. Altered structural connectivity in neonates at genetic risk for schizophrenia: a combined study using morphological and white matter networks.
152. Altered small-world topology of structural brain networks in infants with intrauterine growth restriction and its association with later neurodevelopmental outcome.
153. Analysis of corpus callosum diffusion tensor imaging parameters in infants.
154. Progression of corpus callosum diffusion-tensor imaging values during a period of signal changes consistent with myelination.
155. Therapeutic hypothermia for neonatal encephalopathy results in improved microstructure and metabolism in the deep gray nuclei.
156. Towards the "baby connectome": mapping the structural connectivity of the newborn brain.
157. Fractional anisotropy and mean diffusivity parameters of the brain white matter tracts in preterm infants: reproducibility of region-of-interest measurements.
158. Effect of antenatal growth and prematurity on brain white matter: diffusion tensor study.
159. Quantitative fiber tracking in the corpus callosum and internal capsule reveals microstructural abnormalities in preterm infants at term-equivalent age.
160. A comparison of microstructural maturational changes of the corpus callosum in preterm and full-term children: a diffusion tensor imaging study.
161. Visual functional magnetic resonance imaging of preterm infants.
162. Volumetric MRI and MRS and early motor development of infants born preterm.
163. White matter abnormalities are related to microstructural changes in preterm neonates at term-equivalent age: a diffusion tensor imaging and probabilistic tractography study.
164. Cerebral white matter and neurodevelopment of preterm infants after coagulase-negative staphylococcal sepsis.
165. Impaired brain growth and neurodevelopment in preterm infants with posthaemorrhagic ventricular dilatation.
166. Procedural pain and brain development in premature newborns.
167. Postnatal infection is associated with widespread abnormalities of brain development in premature newborns.
168. Regional cerebral development at term relates to school-age social-emotional development in very preterm children.
169. Quantitative tract-based white matter development from birth to age 2years.
170. Differences in white matter fiber tract development present from 6 to 24 months in infants with autism.
171. Radial structure in the preterm cortex; persistence of the preterm phenotype at term equivalent age?
172. Neural tract development of infants born to methadone-maintained mothers.
173. Prenatal isolated mild ventriculomegaly is associated with persistent ventricle enlargement at ages 1 and 2.
174. Nonlinear microstructural changes in the right superior temporal sulcus and lateral occipitotemporal gyrus between 35 and 43 weeks in the preterm brain.
175. Altered microstructure of white matter except the corpus callosum is independent of prematurity.
176. Neonatal tract-based spatial statistics findings and outcome in preterm infants.
177. Prediction of neurodevelopmental outcome after hypoxic-ischemic encephalopathy treated with hypothermia by diffusion tensor imaging analyzed using tract-based spatial statistics.
178. Regional alterations in cerebral growth exist preoperatively in infants with congenital heart disease.
179. Mapping region-specific longitudinal cortical surface expansion from birth to 2 years of age.
180. Early gray-matter and white-matter concentration in infancy predict later language skills: a whole brain voxel-based morphometry study.
181. The synchronization within and interaction between the default and dorsal attention networks in early infancy.
182. Abnormal cerebral microstructure in premature neonates with congenital heart disease.
183. The development of regional functional connectivity in preterm infants into early childhood.
184. Family poverty affects the rate of human infant brain growth.
185. Morphology and microstructure of subcortical structures at birth: a large-scale Asian neonatal neuroimaging study.
186. Quantification of white matter injury following neonatal stroke with serial DTI.
187. Maturation of corpus callosum anterior midbody is associated with neonatal motor function in eight preterm-born infants.
188. Assessment of structural connectivity in the preterm brain at term equivalent age using diffusion MRI and T2 relaxometry: a network-based analysis.
189. Early development of spatial patterns of power-law frequency scaling in FMRI resting-state and EEG data in the newborn brain.
190. Circulatory insulin-like growth factor-I and brain volumes in relation to neurodevelopmental outcome in very preterm infants.
191. Effect of antenatal growth on brain white matter maturation in preterm infants at term using tract-based spatial statistics.
192. Structural connectivity asymmetry in the neonatal brain.
193. Cortical folding is altered before surgery in infants with congenital heart disease.
194. The influence of preterm birth on the developing thalamocortical connectome.
195. What sleeping babies hear: a functional MRI study of interparental conflict and infants' emotion processing.
196. Effects of white matter injury on resting state fMRI measures in prematurely born infants.
197. Perinatal factors and regional brain volume abnormalities at term in a cohort of extremely low birth weight infants.
198. Pilot randomized trial of hydrocortisone in ventilator-dependent extremely preterm infants: effects on regional brain volumes.
199. Score for neonatal acute physiology-II and neonatal pain predict corticospinal tract development in premature newborns.
200. Gray matter volumetric MRI differences late-preterm and term infants.
201. Microstructural brain and multivoxel spectroscopy in very low birth weight infants related to insulin-like growth factor concentration and early growth.
202. Maternal anxiety and infants' hippocampal development: timing matters.
203. Cerebellar atrophy in childhood arterial ischemic stroke: acute diffusion MRI biomarkers.
204. White matter integrity on fractional anisotropy maps in encephalopathic neonates post hypothermia therapy with normal-appearing MR imaging.
205. Reduced occipital fractional anisotropy on cerebral diffusion tensor imaging in preterm infants with postnatally acquired cytomegalovirus infection.
206. Tractography of white-matter tracts in very preterm infants: a 2-year follow-up study.
207. Hippocampal shape variations at term equivalent age in very preterm infants compared with term controls: perinatal predictors and functional significance at age 7.
208. Magnetic resonance diffusion tensor imaging metrics in perilesional white matter among children with periventricular nodular gray matter heterotopia.
209. DTI values in key white matter tracts from infancy through adolescence.
210. Prenatal maternal depression associates with microstructure of right amygdala in neonates at birth.
211. A machine learning approach to automated structural network analysis: application to neonatal encephalopathy.
212. Normalization of similarity-based individual brain networks from gray matter MRI and its association with neurodevelopment in infants with intrauterine growth restriction.
213. White matter microstructural abnormality in children with hydrocephalus detected by probabilistic diffusion tractography.
214. Brain growth in preterm infants is affected by the degree of growth restriction at birth.
215. Assessment of brain maturation in the preterm infants using diffusion tensor imaging (DTI) and enhanced T2 star weighted angiography (ESWAN).
216. Differences of inter-tract correlations between neonates and children around puberty: a study based on microstructural measurements with DTI.
217. Potential of diffusion tensor MR imaging in the assessment of cognitive impairments in children with periventricular leukomalacia born preterm.
218. Perinatal clinical antecedents of white matter microstructural abnormalities on diffusion tensor imaging in extremely preterm infants.
219. Regional infant brain development: an MRI-based morphometric analysis in 3 to 13 month olds.
220. A DTI-based template-free cortical connectome study of brain maturation.
221. Abnormal brain maturation in preterm neonates associated with adverse developmental outcomes.
222. Quantitative MRI in the very preterm brain: assessing tissue organization and myelination using magnetization transfer, diffusion tensor and T₁ imaging.
223. Language development at 2 years is correlated to brain microstructure in the left superior temporal gyrus at term equivalent age: a diffusion tensor imaging study.
224. Associations between white matter microstructure and infants' working memory.
225. Automatically quantified diffuse excessive high signal intensity on MRI predicts cognitive development in preterm infants.
226. Dorsal and ventral pathways in language development.
227. Frontolimbic neural circuitry at 6 months predicts individual differences in joint attention at 9 months.
228. Breastfeeding and early white matter development: A cross-sectional study.
229. Development of cortical microstructure in the preterm human brain.
230. Radiologic differences in white matter maturation between preterm and full-term infants: TBSS study.
231. Preterm infants' early growth and brain white matter maturation at term age.
232. A multivariate surface-based analysis of the putamen in premature newborns: regional differences within the ventral striatum.
233. Regional characterization of longitudinal DT-MRI to study white matter maturation of the early developing brain.
234. Early brain enlargement and elevated extra-axial fluid in infants who develop autism spectrum disorder.
235. White matter microstructure and atypical visual orienting in 7-month-olds at risk for autism.
236. Functional connectivity in the developing brain: a longitudinal study from 4 to 9months of age.
237. Functional bimodality in the brain networks of preterm and term human newborns.
238. Neonatal neuropsychology: emerging relations of neonatal sensory-motor responses to white matter integrity.
239. Spatial distribution and longitudinal development of deep cortical sulcal landmarks in infants.
240. Mapping longitudinal hemispheric structural asymmetries of the human cerebral cortex from birth to 2 years of age.
241. Mapping longitudinal development of local cortical gyrification in infants from birth to 2 years of age.
242. Longitudinal development of cortical thickness, folding, and fiber density networks in the first 2 years of life.
243. Neonatal cerebral morphometry and later risk of persistent inattention/hyperactivity in children born very preterm.
244. Anatomical correlations of the international 10-20 sensor placement system in infants.
245. Neonatal physiological correlates of near-term brain development on MRI and DTI in very-low-birth-weight preterm infants.
246. Prognostic value of diffusion-weighted imaging summation scores or apparent diffusion coefficient maps in newborns with hypoxic-ischemic encephalopathy.
247. Brain microstructural development at near-term age in very-low-birth-weight preterm infants: an atlas-based diffusion imaging study.
248. Temporal resolvability analysis of macroscopic morphological development in neonatal cerebral magnetic resonance images.
249. White matter development and early cognition in babies and toddlers.
250. Reduced brain resting-state network specificity in infants compared with adults.
251. Brain differences in infants at differential genetic risk for late-onset Alzheimer disease: a cross-sectional imaging study.
252. Reliability and repeatability of quantitative tractography methods for mapping structural white matter connectivity in preterm and term infants at term-equivalent age.
253. Microstructural brain development between 30 and 40 weeks corrected age in a longitudinal cohort of extremely preterm infants.
254. Alterations in brain structure and neurodevelopmental outcome in preterm infants hospitalized in different neonatal intensive care unit environments.
255. Maternal obesity and increased risk for autism and developmental delay among very preterm infants.
256. Neural specialization for speech in the first months of life.
257. Multivariate longitudinal shape analysis of human lateral ventricles during the first twenty-four months of life.
258. Structural network analysis of brain development in young preterm neonates.
259. Body growth and brain development in premature babies: an MRI study.
260. Preterm birth affects the developmental synergy between cortical folding and cortical connectivity observed on multimodal MRI.
261. Rich-club organization of the newborn human brain.
262. Age-related changes in tissue signal properties within cortical areas important for word understanding in 12- to 19-month-old infants.
263. Diffuse excessive high signal intensity in low-risk preterm infants at term-equivalent age does not predict outcome at 1 year: a prospective study.
264. Whole-brain mapping of structural connectivity in infants reveals altered connection strength associated with growth and preterm birth.
265. Assessing sequence and relationship of regional maturation in corpus callosum and internal capsule in preterm and term newborns by diffusion-tensor imaging.
266. Differential developmental trajectories of magnetic susceptibility in human brain gray and white matter over the lifespan.
267. Normal centrolineal myelination of the callosal splenium reflects the development of the cortical origin and size of its commissural fibers.
268. Correlation between fractional anisotropy and motor outcomes in one-year-old infants with periventricular brain injury.
269. Sequential cranial ultrasound and cerebellar diffusion weighted imaging contribute to the early prognosis of neurodevelopmental outcome in preterm infants.
270. Effect of thyroxine on brain microstructure in extremely premature babies: magnetic resonance imaging findings in the TIPIT study.
271. White matter NAA/Cho and Cho/Cr ratios at MR spectroscopy are predictive of motor outcome in preterm infants.
272. Diffusion-weighted imaging and magnetic resonance proton spectroscopy following preterm birth.
273. The impact of prenatal and neonatal infection on neurodevelopmental outcomes in very preterm infants.
274. Choice of diffusion tensor estimation approach affects fiber tractography of the fornix in preterm brain.
275. Do maternal opioids reduce neonatal regional brain volumes? A pilot study.
276. Development of the optic radiations and visual function after premature birth.
277. Unmyelinated white matter loss in the preterm brain is associated with early increased levels of end-tidal carbon monoxide.
278. Brain volume and neurobehavior in newborns with complex congenital heart defects.
279. Assessment of iron deposition and white matter maturation in infant brains by using enhanced T2 star weighted angiography (ESWAN): R2* versus phase values.
280. Altered microstructural connectivity of the superior and middle cerebellar peduncles are related to motor dysfunction in children with diffuse periventricular leucomalacia born preterm: a DTI tractography study.
281. Longitudinal growth and morphology of the hippocampus through childhood: Impact of prematurity and implications for memory and learning.
282. Accelerated myelination along fiber tracts in patients with hemimegalencephaly.
283. Preterm nutritional intake and MRI phenotype at term age: a prospective observational study.
284. Common variants in psychiatric risk genes predict brain structure at birth.
285. Prenatal cocaine effects on brain structure in early infancy.
286. Intersubject variability of and genetic effects on the brain's functional connectivity during infancy.
287. Structural growth trajectories and rates of change in the first 3 months of infant brain development.
288. Development of thalamocortical connectivity during infancy and its cognitive correlations.
289. White matter microstructure is influenced by extremely preterm birth and neonatal respiratory factors.
290. Serial diffusion tensor images during infancy and their relationship to neuromotor outcomes in preterm infants.
291. Sex differences in outcome and associations with neonatal brain morphology in extremely preterm children.
292. Neonatal encephalopathic cerebral injury in South India assessed by perinatal magnetic resonance biomarkers and early childhood neurodevelopmental outcome.
293. Common genetic variants and risk of brain injury after preterm birth.
294. Differential vulnerability of gray matter and white matter to intrauterine growth restriction in preterm infants at 12 months corrected age.
295. White matter injury in newborns with congenital heart disease: a diffusion tensor imaging study.
296. GABA, resting-state connectivity and the developing brain.
297. Impact of sex and gonadal steroids on neonatal brain structure.
298. Gestational age and neonatal brain microstructure in term born infants: a birth cohort study.
299. Isolated mild white matter signal changes in preterm infants: a regional approach for comparison of cranial ultrasound and MRI findings.
300. Early MRI in term infants with perinatal hypoxic-ischaemic brain injury: interobserver agreement and MRI predictors of outcome at 2 years.
301. Morphological features of the neonatal brain support development of subsequent cognitive, language, and motor abilities.
302. Validation of a brain MRI relaxometry protocol to measure effects of preterm birth at a flexible postnatal age.
303. Diffusion tensor imaging in extremely low birth weight infants managed with hypercapnic vs. normocapnic ventilation.
304. Role of diffusion tensor imaging as an independent predictor of cognitive and language development in extremely low-birth-weight infants.
305. Impaired white matter development in extremely low-birth-weight infants with previous brain hemorrhage.
306. Characterization of microstructural injury: a novel approach in infant abusive head trauma-initial experience.
307. An allometric scaling relationship in the brain of preterm infants.
308. Modeling healthy male white matter and myelin development: 3 through 60months of age.
309. Frequency of spontaneous BOLD signal shifts during infancy and correlates with cognitive performance.
310. Specialization and integration of functional thalamocortical connectivity in the human infant.
311. Maternal sensitivity, infant limbic structure volume and functional connectivity: a preliminary study.
312. Prenatal maternal depression alters amygdala functional connectivity in 6-month-old infants.
313. Early Brain Activity Relates to Subsequent Brain Growth in Premature Infants.
314. Accurate age classification of 6 and 12 month-old infants based on resting-state functional connectivity magnetic resonance imaging data.
315. Thalamocortical Connectivity Predicts Cognition in Children Born Preterm.
316. fMRI reveals neural activity overlap between adult and infant pain.
317. Neonatal MRI is associated with future cognition and academic achievement in preterm children.
318. Dynamic Development of Regional Cortical Thickness and Surface Area in Early Childhood.
319. Spatial Patterns, Longitudinal Development, and Hemispheric Asymmetries of Cortical Thickness in Infants from Birth to 2 Years of Age.
320. Spatiotemporal patterns of cortical fiber density in developing infants, and their relationship with cortical thickness.
321. Aberrant Executive and Frontoparietal Functional Connectivity in Very Preterm Infants With Diffuse White Matter Abnormalities.
322. A semi-supervised Support Vector Machine model for predicting the language outcomes following cochlear implantation based on pre-implant brain fMRI imaging.
323. Brain structural connectivity increases concurrent with functional improvement: evidence from diffusion tensor MRI in children with cerebral palsy during therapy.
324. Functional magnetic resonance imaging can be used to explore tactile and nociceptive processing in the infant brain.
325. Early Postnatal Myelin Content Estimate of White Matter via T1w/T2w Ratio.
326. Enhanced nutrient supply to very low birth weight infants is associated with improved white matter maturation and head growth.
327. Neonatal DTI early after birth predicts motor outcome in preterm infants with periventricular hemorrhagic infarction.
328. Regional impairments of cortical folding in premature infants.
329. Functional properties of resting state networks in healthy full-term newborns.
330. High frequency functional brain networks in neonates revealed by rapid acquisition resting state fMRI.
331. Development of human brain structural networks through infancy and childhood.
332. The Neonatal Connectome During Preterm Brain Development.
333. Development of human brain cortical network architecture during infancy.
334. Adaptive mechanisms of developing brain: cerebral lateralization in the prematurely-born.
335. Corticospinal Tract Injury Precedes Thalamic Volume Reduction in Preterm Infants with Cystic Periventricular Leukomalacia.
336. Early life stress is associated with default system integrity and emotionality during infancy.
337. Atypical processing of voice sounds in infants at risk for autism spectrum disorder.
338. Neonatal morphine exposure in very preterm infants-cerebral development and outcomes.
339. Brain-derived neurotrophic factor (BDNF) Val66Met polymorphism influences the association of the methylome with maternal anxiety and neonatal brain volumes.
340. Brain Growth Gains and Losses in Extremely Preterm Infants at Term.
341. Developmental synergy between thalamic structure and interhemispheric connectivity in the visual system of preterm infants.
342. Functional Network Development During the First Year: Relative Sequence and Socioeconomic Correlations.
343. White matter tract integrity and developmental outcome in newborn infants with hypoxic-ischemic encephalopathy treated with hypothermia.
344. Neonatal neurobehavior after therapeutic hypothermia for hypoxic ischemic encephalopathy.
345. Impaired Global and Regional Cerebral Perfusion in Newborns with Complex Congenital Heart Disease.
346. A DTI-based tractography study of effects on brain structure associated with prenatal alcohol exposure in newborns.
347. Abnormal development of thalamic microstructure in premature neonates with congenital heart disease.
348. Therapeutic hypothermia modifies perinatal asphyxia-induced changes of the corpus callosum and outcome in neonates.
349. Evolution of Apparent Diffusion Coefficient and Fractional Anisotropy in the Cerebrum of Asphyxiated Newborns Treated with Hypothermia over the First Month of Life.
350. Neurodevelopmental outcome at 36 months in very low birth weight premature infants with MR diffuse excessive high signal intensity (DEHSI) of cerebral white matter.
351. Increased Brain Perfusion Persists over the First Month of Life in Term Asphyxiated Newborns Treated with Hypothermia: Does it Reflect Activated Angiogenesis?
352. Apparent diffusion coefficient scalars correlate with near-infrared spectroscopy markers of cerebrovascular autoregulation in neonates cooled for perinatal hypoxic-ischemic injury.
353. Hippocampal Malrotation Is Associated With Prolonged Febrile Seizures: Results of the FEBSTAT Study.
354. A pilot randomized trial of high-dose caffeine therapy in preterm infants.
355. Injury to the Cerebellum in Term Asphyxiated Newborns Treated with Hypothermia.
356. Accelerated corpus callosum development in prematurity predicts improved outcome.
357. Characterizing longitudinal white matter development during early childhood.
358. Reduced thalamic volume in preterm infants is associated with abnormal white matter metabolism independent of injury.
359. Low-grade intraventricular hemorrhage disrupts cerebellar white matter in preterm infants: evidence from diffusion tensor imaging.
360. Antenatal maternal anxiety predicts variations in neural structures implicated in anxiety disorders in newborns.
361. Cerebral maturation in the early preterm period-A magnetization transfer and diffusion tensor imaging study using voxel-based analysis.
362. Maternal Postsecondary Education Associated With Improved Cerebellar Growth After Preterm Birth.
363. Quantitative tract-based white matter heritability in twin neonates.
364. Cortical maturation and myelination in healthy toddlers and young children.
365. Different functional neural substrates for good and poor language outcome in autism.
366. Neuroimaging identifies increased manganese deposition in infants receiving parenteral nutrition.
367. Morphological features of the neonatal brain following exposure to regional anesthesia during labor and delivery.
368. Prenatal drug exposure affects neonatal brain functional connectivity.
369. Visual fixation in human newborns correlates with extensive white matter networks and predicts long-term neurocognitive development.
370. Quantitative magnetic resonance imaging evidence for altered structural remodeling of the temporal lobe in West syndrome.
371. Maternal adiposity negatively influences infant brain white matter development.
372. Development of Cortical Morphology Evaluated with Longitudinal MR Brain Images of Preterm Infants.
373. Regional differences in fiber tractography predict neurodevelopmental outcomes in neonates with infantile Krabbe disease.
374. Altered corpus callosum morphology associated with autism over the first 2 years of life.
375. Consistent anterior-posterior segregation of the insula during the first 2 years of life.
376. COMT haplotypes modulate associations of antenatal maternal anxiety and neonatal cortical morphology.
377. Deep grey matter growth predicts neurodevelopmental outcomes in very preterm children.
378. Developmental synchrony of thalamocortical circuits in the neonatal brain.
379. Tract-based spatial statistics to assess the neuroprotective effect of early erythropoietin on white matter development in preterm infants.
380. Development of the Corticospinal and Callosal Tracts from Extremely Premature Birth up to 2 Years of Age.
381. Tract-Based Spatial Statistics in Preterm-Born Neonates Predicts Cognitive and Motor Outcomes at 18 Months.
382. A study of the effects of prenatal alcohol exposure on white matter microstructural integrity at birth.
383. Comparing tract-based spatial statistics and manual region-of-Interest labeling as diffusion analysis methods to detect white matter abnormalities in infants with hypoxic-Ischemic encephalopathy.
384. Maturation of preterm newborn brains: a fMRI-DTI study of auditory processing of linguistic stimuli and white matter development.
385. BOLD Response Selective to Flow-Motion in Very Young Infants.
386. Diffusion Tensor Imaging Provides Evidence of Possible Axonal Overconnectivity in Frontal Lobes in Autism Spectrum Disorder Toddlers.
387. Probabilistic maps of the white matter tracts with known associated functions on the neonatal brain atlas: Application to evaluate longitudinal developmental trajectories in term-born and preterm-born infants.
388. Association between preterm brain injury and exposure to chorioamnionitis during fetal life.
389. Implications of newborn amygdala connectivity for fear and cognitive development at 6-months-of-age.
390. Functional and structural connectivity of the visual system in infants with perinatal brain injury.
391. Maternal prenatal iron status and tissue organization in the neonatal brain.
392. fMRI as a Preimplant Objective Tool to Predict Postimplant Oral Language Outcomes in Children with Cochlear Implants.
393. Perinatal MRI diffusivity is related to early assessment of motor performance in preterm neonates.
394. Brain functional network connectivity development in very preterm infants: The first six months.
395. Correlating early motor skills to white matter abnormalities in preterm infants using diffusion tensor imaging.
396. Sex-Specific Alterations of White Matter Developmental Trajectories in Infants With Prenatal Exposure to Methamphetamine and Tobacco.
397. Delayed early developmental trajectories of white matter tracts of functional pathways in preterm-born infants: Longitudinal diffusion tensor imaging data.
398. Cortical folding of the preterm brain: a longitudinal analysis of extremely preterm born neonates using spectral matching.
399. White matter maturation profiles through early childhood predict general cognitive ability.
400. Gray Matter Growth Is Accompanied by Increasing Blood Flow and Decreasing Apparent Diffusion Coefficient during Childhood.
401. Default mode network abnormalities in children with autism spectrum disorder detected by resting-state functional magnetic resonance imaging.
402. Left hemisphere structural connectivity abnormality in pediatric hydrocephalus patients following surgery.
403. Hindbrain regional growth in preterm newborns and its impairment in relation to brain injury.
404. Frequency of Spontaneous BOLD Signal Differences between Moderate and Late Preterm Newborns and Term Newborns.
405. Alterations in amygdala-prefrontal circuits in infants exposed to prenatal maternal depression.
406. Alcohol exposure in utero is associated with decreased gray matter volume in neonates.
407. Interhemispheric Functional Brain Connectivity in Neonates with Prenatal Alcohol Exposure: Preliminary Findings.
408. Biomechanical Analysis of Normal Brain Development during the First Year of Life Using Finite Strain Theory.
409. Structural Brain Network Reorganization and Social Cognition Related to Adverse Perinatal Condition from Infancy to Early Adolescence.
410. Altered resting-state whole-brain functional networks of neonates with intrauterine growth restriction.
411. Resting-State Network Complexity and Magnitude Are Reduced in Prematurely Born Infants.
412. Thalamocortical functional connectivity and behavioral disruptions in neonates with prenatal cocaine exposure.
413. Longitudinal Regional Brain Development and Clinical Risk Factors in Extremely Preterm Infants.
414. Brain Volumes at Term-Equivalent Age Are Associated with 2-Year Neurodevelopment in Moderate and Late Preterm Children.
415. Evolution of T1 Relaxation, ADC, and Fractional Anisotropy during Early Brain Maturation: A Serial Imaging Study on Preterm Infants.
416. Creatine, Glutamine plus Glutamate, and Macromolecules Are Decreased in the Central White Matter of Premature Neonates around Term.
417. Effects of Posthemorrhagic Ventricular Dilatation in the Preterm Infant on Brain Volumes and White Matter Diffusion Variables at Term-Equivalent Age.
418. Examining the relationships between cortical maturation and white matter myelination throughout early childhood.
419. Neonatal diffusion tensor brain imaging predicts later motor outcome in preterm neonates with white matter abnormalities.
420. Smaller Cerebellar Growth and Poorer Neurodevelopmental Outcomes in Very Preterm Infants Exposed to Neonatal Morphine.
421. Exploring the Early Organization and Maturation of Linguistic Pathways in the Human Infant Brain.
422. Cockayne syndrome: a diffusion tensor imaging and volumetric study.
423. Cerebellar Development in Preterm Infants at Term-Equivalent Age Is Impaired after Low-Grade Intraventricular Hemorrhage.
424. Initial Application of Diffusional Kurtosis Imaging in Evaluating Brain Development of Healthy Preterm Infants.
425. Altered Amygdala Development and Fear Processing in Prematurely Born Infants.
426. Regional Microstructural and Volumetric Magnetic Resonance Imaging (MRI) Abnormalities in the Corpus Callosum of Neonates With Congenital Heart Defect Undergoing Cardiac Surgery.
427. The motor and visual networks in preterm infants: An fMRI and DTI study.
428. Regional white matter development in very preterm infants: perinatal predictors and early developmental outcomes.
429. Diffusion tensor imaging detects early brain microstructure changes before and after ventriculoperitoneal shunt in children with high intracranial pressure hydrocephalus.
430. Cortical Gray and Adjacent White Matter Demonstrate Synchronous Maturation in Very Preterm Infants.
431. Diffusion tensor imaging and MR spectroscopy of microstructural alterations and metabolite concentration changes in the auditory neural pathway of pediatric congenital sensorineural hearing loss patients.
432. Developmental process of the arcuate fasciculus from infancy to adolescence: a diffusion tensor imaging study.
433. Diffusion tensor imaging study of pediatric patients with congenital hydrocephalus: 1-year postsurgical outcomes.
434. Third Trimester Brain Growth in Preterm Infants Compared With In Utero Healthy Fetuses.
435. White matter disruption is associated with persistent seizures in tuberous sclerosis complex.
436. Motor and cortico-striatal-thalamic connectivity alterations in intrauterine growth restriction.
437. Epigenomic profiling of preterm infants reveals DNA methylation differences at sites associated with neural function.
438. Development of human white matter fiber pathways: From newborn to adult ages.
439. Relation between clinical risk factors, early cortical changes, and neurodevelopmental outcome in preterm infants.
440. Brain Volumes at Term-Equivalent Age in Preterm Infants: Imaging Biomarkers for Neurodevelopmental Outcome through Early School Age.
441. Quantification of structural changes in the corpus callosumin children with profound hypoxic-ischaemic brain injury.
442. Examination of the Pattern of Growth of Cerebral Tissue Volumes From Hospital Discharge to Early Childhood in Very Preterm Infants.
443. Delayed cortical gray matter development in neonates with severe congenital heart disease.
444. Longitudinal cerebellar growth following very preterm birth.
445. Midazolam dose correlates with abnormal hippocampal growth and neurodevelopmental outcome in preterm infants.
446. Effect of socioeconomic status (SES) disparity on neural development in female African-American infants at age 1 month.
447. Preterm birth alters neonatal, functional rich club organization.
448. Prenatal stress alters amygdala functional connectivity in preterm neonates.
449. Antenatal depression, treatment with selective serotonin reuptake inhibitors, and neonatal brain structure: A propensity-matched cohort study.
450. Diffusion tensor imaging-based assessment of white matter tracts and visual-motor outcomes in very preterm neonates.
451. Differences in brain functional connectivity at resting state in neonates born to healthy obese or normal-weight mothers.
452. Longitudinal Study of the Emerging Functional Connectivity Asymmetry of Primary Language Regions during Infancy.
453. Moderate and late preterm infants exhibit widespread brain white matter microstructure alterations at term-equivalent age relative to term-born controls.
454. Early postnatal docosahexaenoic acid levels and improved preterm brain development.
455. White Matter Abnormality Correlates with Developmental and Seizure Outcomes in West Syndrome of Unknown Etiology.
456. Altered white matter and cortical structure in neonates with antenatally diagnosed isolated ventriculomegaly.
457. White Matter Microstructural Integrity and Neurobehavioral Outcome of HIV-Exposed Uninfected Neonates.
458. Possible relationship between common genetic variation and white matter development in a pilot study of preterm infants.
459. Exploring the role of white matter connectivity in cortex maturation.
460. The Emergence of Network Inefficiencies in Infants With Autism Spectrum Disorder.
461. White Matter Alterations in Infants at Risk for Developmental Dyslexia.
462. Poor Brain Growth in Extremely Preterm Neonates Long Before the Onset of Autism Spectrum Disorder Symptoms.
463. Heterogeneous increases of regional cerebral blood flow during preterm brain development: Preliminary assessment with pseudo-continuous arterial spin labeled perfusion MRI.
464. Functional neuroimaging of high-risk 6-month-old infants predicts a diagnosis of autism at 24 months of age.
465. Early development of structural networks and the impact of prematurity on brain connectivity.
466. Detection and Growth Pattern of Arcuate Fasciculus from Newborn to Adult.
467. Change-point analysis data of neonatal diffusion tensor MRI in preterm and term-born infants.
468. Mapping the critical gestational age at birth that alters brain development in preterm-born infants using multi-modal MRI.
469. Effect of Autologous Cord Blood Infusion on Motor Function and Brain Connectivity in Young Children with Cerebral Palsy: A Randomized, Placebo-Controlled Trial.
470. Diffusion tensor imaging assesses white matter injury in neonates with hypoxic-ischemic encephalopathy.
471. Mother-infant interactions and regional brain volumes in infancy: an MRI study.
472. Investigating the maturation of microstructure and radial orientation in the preterm human cortex with diffusion MRI.
473. Microstructure of the Default Mode Network in Preterm Infants.
474. Altered Brain Functional Activity in Infants with Congenital Bilateral Severe Sensorineural Hearing Loss: A Resting-State Functional MRI Study under Sedation.
475. Towards a unified analysis of brain maturation and aging across the entire lifespan: A MRI analysis.
476. Neonatal neural networks predict children behavioral profiles later in life.
477. White matter maturation in the neonatal brain is predictive of school age cognitive capacities in children born very preterm.
478. Quantifying cortical development in typically developing toddlers and young children, 1-6 years of age.
479. Cortical multisensory connectivity is present near birth in humans.
480. Adult-like processing of naturalistic sounds in auditory cortex by 3- and 9-month old infants.
481. Emergence of a hierarchical brain during infancy reflected by stepwise functional connectivity.
482. Cerebello-cerebral connectivity in the developing brain.
483. Using Functional Connectivity Magnetic Resonance Imaging to Measure Brain Connectivity in Preterm Infants.
484. Migration Pathways of Thalamic Neurons and Development of Thalamocortical Connections in Humans Revealed by Diffusion MR Tractography.
485. Ventricular shape and relative position abnormalities in preterm neonates.
486. Genome-wide association analysis identifies common variants influencing infant brain volumes.
487. Functional thalamocortical connectivity development and alterations in preterm infants during the neonatal period.
488. Prematurity and brain perfusion: Arterial spin labeling MRI.
489. Early Imaging and Adverse Neurodevelopmental Outcome in Asphyxiated Newborns Treated With Hypothermia.
490. High-angular resolution diffusion imaging tractography of cerebellar pathways from newborns to young adults.
491. Diffusion Kurtosis Imaging of Microstructural Alterations in the Brains of Paediatric Patients with Congenital Sensorineural Hearing Loss.
492. Newborn insula gray matter volume is prospectively associated with early life adiposity gain.
493. Comparison of fractional anisotropy and apparent diffusion coefficient among hypoxic ischemic encephalopathy stages 1, 2, and 3 and with nonasphyxiated newborns in 18 areas of brain.
494. Altered brain function in new onset childhood acute lymphoblastic leukemia before chemotherapy: A resting-state fMRI study.
495. Development of the Corpus Callosum: An MRI Study.
496. MRI Differences Associated with Intrauterine Growth Restriction in Preterm Infants.
497. Longitudinal change in white matter in preterm infants without magnetic resonance imaging abnormalities: Assessment of serial diffusion tensor imaging and their relationship to neurodevelopmental outcomes.
498. Changes in brain microstructure during infancy and childhood using clinical feasible ADC-maps.
499. Toddlers later diagnosed with autism exhibit multiple structural abnormalities in temporal corpus callosum fibers.
500. Heavy Prenatal Alcohol Exposure is Related to Smaller Corpus Callosum in Newborn MRI Scans.
501. Can cerebellar and brainstem apparent diffusion coefficient (ADC) values predict neuromotor outcome in term neonates with hypoxic-ischemic encephalopathy (HIE) treated with hypothermia?
502. Association between corpus callosum development on magnetic resonance imaging and diffusion tensor imaging, and neurodevelopmental outcome in neonates born very preterm.
503. Neonatal Amygdala Functional Connectivity at Rest in Healthy and Preterm Infants and Early Internalizing Symptoms.
504. Antenatal exposure to antidepressants is associated with altered brain development in very preterm-born neonates.
505. Cerebellar Microstructural Organization is Altered by Complications of Premature Birth: A Case-Control Study.
506. Alteration in the number and integrity of white matter tracts in the preterm: A quantitative diffusion tensor imaging and diffusion fibre tractography in children.
507. White Matter Volume Predicts Language Development in Congenital Heart Disease.
508. Language ability in preterm children is associated with arcuate fasciculi microstructure at term.
509. A latent measure explains substantial variance in white matter microstructure across the newborn human brain.
510. Regional differences in interhemispheric structural fibers in healthy, term infants.
511. Twin-singleton developmental study of brain white matter anatomy.
512. Effects of Antenatal Maternal Depressive Symptoms and Socio-Economic Status on Neonatal Brain Development are Modulated by Genetic Risk.
513. Early Development of Functional Network Segregation Revealed by Connectomic Analysis of the Preterm Human Brain.
514. Longitudinal Study of White Matter Development and Outcomes in Children Born Very Preterm.
515. Characterization of Extensive Microstructural Variations Associated with Punctate White Matter Lesions in Preterm Neonates.
516. Early detection of neonatal hypoxic-ischemic white matter injury: an MR diffusion tensor imaging study.
517. White Matter Injury and General Movements in High-Risk Preterm Infants.
518. Quantitative assessment of white matter injury in preterm neonates: Association with outcomes.
519. Structural and Maturational Covariance in Early Childhood Brain Development.
520. Impact of Demographic and Obstetric Factors on Infant Brain Volumes: A Population Neuroscience Study.
521. Complementary cortical gray and white matter developmental patterns in healthy, preterm neonates.
522. Resting-state fMRI in sleeping infants more closely resembles adult sleep than adult wakefulness.
523. Splenium development and early spoken language in human infants.
524. Asynchronous Development of Cerebellar, Cerebello-Cortical, and Cortico-Cortical Functional Networks in Infancy, Childhood, and Adulthood.
525. Joint Attention and Brain Functional Connectivity in Infants and Toddlers.
526. Mapping White Matter Microstructure in the One Month Human Brain.
527. Neonatal basal ganglia and thalamic volumes: very preterm birth and 7-year neurodevelopmental outcomes.
528. Common and heritable components of white matter microstructure predict cognitive function at 1 and 2 y.
529. A novel maturation index based on neonatal diffusion tensor imaging reflects typical perinatal white matter development in humans.
530. Subcortical Brain and Behavior Phenotypes Differentiate Infants With Autism Versus Language Delay.
531. Punctate White Matter Lesions Associated With Altered Brain Development And Adverse Motor Outcome In Preterm Infants.
532. Early brain development in infants at high risk for autism spectrum disorder.
533. Neural circuitry at age 6 months associated with later repetitive behavior and sensory responsiveness in autism.
534. Gestational Age at Birth and Brain White Matter Development in Term-Born Infants and Children.
535. Severe retinopathy of prematurity predicts delayed white matter maturation and poorer neurodevelopment.
536. Maternal Adiposity Influences Neonatal Brain Functional Connectivity.
537. The Impact of Caregiving on the Association Between Infant Emotional Behavior and Resting State Neural Network Functional Topology.
538. Cerebellar peduncle injury predicts motor impairments in preterm infants: A quantitative tractography study at term-equivalent age.
539. Randomized controlled trial of brain specific fatty acid supplementation in pregnant women increases brain volumes on MRI scans of their newborn infants.
540. Caffeine for apnea of prematurity and brain development at 11 years of age.
541. Multidelay Arterial Spin-Labeling MRI in Neonates and Infants: Cerebral Perfusion Changes during Brain Maturation.
542. Volume-Based Analysis of 6-Month-Old Infant Brain MRI for Autism Biomarker Identification and Early Diagnosis.
543. Structural network topology correlates of microstructural brain dysmaturation in term infants with congenital heart disease.
544. Somatotopic Mapping of the Developing Sensorimotor Cortex in the Preterm Human Brain.
545. Associations Between Brain Structure and Connectivity in Infants and Exposure to Selective Serotonin Reuptake Inhibitors During Pregnancy.
546. Conventional MRI scan and DTI imaging show more severe brain injury in neonates with hypoxic-ischemic encephalopathy and seizures.
547. A lateral-to-mesial organization of human ventral visual cortex at birth.
548. Why does language not emerge until the second year?
549. Quantitative Apparent Diffusion Coefficient Mapping May Predict Seizure Onset in Children With Sturge-Weber Syndrome.
550. Maternal IL-6 during pregnancy can be estimated from newborn brain connectivity and predicts future working memory in offspring.
551. Visual-motor integration and fine motor skills at 6½ years of age and associations with neonatal brain volumes in children born extremely preterm in Sweden: a population-based cohort study.
552. Disruption to functional networks in neonates with perinatal brain injury predicts motor skills at 8 months.
553. Multiple Postnatal Infections in Newborns Born Preterm Predict Delayed Maturation of Motor Pathways at Term-Equivalent Age with Poorer Motor Outcomes at 3 Years.
554. Auditory structural connectivity in preterm and healthy term infants during the first postnatal year.
555. Differential Rates of Perinatal Maturation of Human Primary and Nonprimary Auditory Cortex.
556. Early Procedural Pain Is Associated with Regionally-Specific Alterations in Thalamic Development in Preterm Neonates.
557. Effects of early nutrition and growth on brain volumes, white matter microstructure, and neurodevelopmental outcome in preterm newborns.
558. FKBP5 Moderates the Association between Antenatal Maternal Depressive Symptoms and Neonatal Brain Morphology.
559. Cortical Functional Connectivity Evident After Birth and Behavioral Inhibition at Age 2.
560. MRI Changes in the Thalamus and Basal Ganglia of Full-Term Neonates with Perinatal Asphyxia.
561. Prenatal methadone exposure is associated with altered neonatal brain development.
562. The influence of the descending pain modulatory system on infant pain-related brain activity.
563. Genetic influences on neonatal cortical thickness and surface area.
564. Body length and occipitofrontal circumference may be good indicators of neurodevelopment in very low birthweight infants - secondary publication.
565. Association of Histologic Chorioamnionitis With Perinatal Brain Injury and Early Childhood Neurodevelopmental Outcomes Among Preterm Neonates.
566. Brain growth in the NICU: critical periods of tissue-specific expansion.
567. Quantitative susceptibility map analysis in preterm neonates with germinal matrix-intraventricular hemorrhage.
568. T2 Relaxometry MRI Predicts Cerebral Palsy in Preterm Infants.
569. Thalamic volume and dimensions on MRI in the pediatric population: Normative values and correlations: (A cross sectional study).
570. Intergenerational Effect of Maternal Exposure to Childhood Maltreatment on Newborn Brain Anatomy.
571. Seizure Severity Is Correlated With Severity of Hypoxic-Ischemic Injury in Abusive Head Trauma.
572. Cerebellar volume as an imaging marker of development in infants with tuberous sclerosis complex.
573. Dynamic patterns of cortical expansion during folding of the preterm human brain.
574. Prediction of cognitive and motor development in preterm children using exhaustive feature selection and cross-validation of near-term white matter microstructure.
575. Human amygdala functional network development: A cross-sectional study from 3 months to 5 years of age.
576. Resting state signal latency predicts laterality in pediatric medically refractory temporal lobe epilepsy.
577. Rapid high resolution T1 mapping as a marker of brain development: Normative ranges in key regions of interest.
578. MR Imaging of hypoxic ischemic encephalopathy - Distribution Patterns and ADC value correlations.
579. Association of Prenatal Maternal Depression and Anxiety Symptoms With Infant White Matter Microstructure.
580. Longitudinal Preterm Cerebellar Volume: Perinatal and Neurodevelopmental Outcome Associations.
581. Early human brain development: insights into macroscale connectome wiring.
582. fMRI-based Neuronal Response to New Odorants in the Newborn Brain.
583. Altered functional network connectivity in preterm infants: antecedents of cognitive and motor impairments?
584. Diffusion MRI parameters of corpus callosum and corticospinal tract in neonates: Comparison between region-of-interest and whole tract averaged measurements.
585. Postnatal Microstructural Developmental Trajectory of Corpus Callosum Subregions and Relationship to Clinical Factors in Very Preterm Infants.
586. Advanced diffusion imaging for assessing normal white matter development in neonates and characterizing aberrant development in congenital heart disease.
587. Development of White Matter Circuitry in Infants With Fragile X Syndrome.
588. Functional Brain Parcellations of the Infant Brain and the Associated Developmental Trends.
589. Characteristic phase distribution in the white matter of infants on phase difference enhanced imaging.
590. Mild cerebellar injury does not significantly affect cerebral white matter microstructural organization and neurodevelopmental outcome in a contemporary cohort of preterm infants.
591. Regional microstructural organization of the cerebral cortex is affected by preterm birth.
592. Procedural pain and oral glucose in preterm neonates: brain development and sex-specific effects.
593. Nutrient Intake in the First Two Weeks of Life and Brain Growth in Preterm Neonates.
594. Severe retinopathy of prematurity is associated with reduced cerebellar and brainstem volumes at term and neurodevelopmental deficits at 2 years.
595. Walking, Gross Motor Development, and Brain Functional Connectivity in Infants and Toddlers.
596. Early prediction of cognitive deficits in very preterm infants using functional connectome data in an artificial neural network framework.
597. Changes in brain morphology and microstructure in relation to early brain activity in extremely preterm infants.
598. Fixel-based analysis reveals alterations is brain microstructure and macrostructure of preterm-born infants at term equivalent age.
599. Cytotoxic edema at onset in West syndrome of unknown etiology: A longitudinal diffusion tensor imaging study.
600. Investigation of brain structure in the 1-month infant.
601. Right but not left hemispheric discrimination of faces in infancy.
602. Cerebral White Matter Maturation Patterns in Preterm Infants: An MRI T2 Relaxation Anisotropy and Diffusion Tensor Imaging Study.
603. The Cerebellar-Cerebral Microstructure Is Disrupted at Multiple Sites in Very Preterm Infants with Cerebellar Haemorrhage.
604. Probabilistic tractography-based thalamic parcellation in healthy newborns and newborns with congenital heart disease.
605. Prenatal methamphetamine exposure is associated with corticostriatal white matter changes in neonates.
606. Prenatal methamphetamine exposure is associated with reduced subcortical volumes in neonates.
607. Postnatal polyunsaturated fatty acids associated with larger preterm brain tissue volumes and better outcomes.
608. The effects of mild germinal matrix-intraventricular haemorrhage on the developmental white matter microstructure of preterm neonates: a DTI study.
609. Maternal Systemic Interleukin-6 During Pregnancy Is Associated With Newborn Amygdala Phenotypes and Subsequent Behavior at 2 Years of Age.
610. Retinopathy of Prematurity and Bronchopulmonary Dysplasia are Independent Antecedents of Cortical Maturational Abnormalities in Very Preterm Infants.
611. Machine Learning Assisted MRI Characterization for Diagnosis of Neonatal Acute Bilirubin Encephalopathy.
612. Individual differences in neonatal white matter are associated with executive function at 3 years of age.
613. Detection of occult abnormalities in the deep gray matter nuclei of neonates with punctate white matter lesions by magnetic resonance spectroscopy.
614. Maternal pomegranate juice intake and brain structure and function in infants with intrauterine growth restriction: A randomized controlled pilot study.
615. Infant Brain Structural MRI Analysis in the Context of Thoracic Non-cardiac Surgery and Critical Care.
616. Altered Functional Brain Network Integration, Segregation, and Modularity in Infants Born Very Preterm at Term-Equivalent Age.
617. Reduced structural brain asymmetry during neonatal life is potentially related to autism spectrum disorders in children born extremely preterm.
618. Aberrant myelination in patients with Sturge-Weber syndrome analyzed using synthetic quantitative magnetic resonance imaging.
619. Diffusion Tensor Imaging Analysis of White Matter Microstructural Integrity in Infants With Retinopathy of Prematurity.
620. Altered neonatal white and gray matter microstructure is associated with neurodevelopmental impairments in very preterm infants with high-grade brain injury.
621. Ultrasound Predicts White Matter Integrity after Hypothermia Therapy in Neonatal Hypoxic-Ischemic Injury.
622. Music in premature infants enhances high-level cognitive brain networks.
623. Failure to attune to language predicts autism in high risk infants.
624. Correlation Between White Matter Injury Identified by Neonatal Diffusion Tensor Imaging and Neurodevelopmental Outcomes Following Term Neonatal Asphyxia and Therapeutic Hypothermia: An Exploratory Pilot Study.
625. Alterations of structural and functional connectivity in profound sensorineural hearing loss infants within an early sensitive period: A combined DTI and fMRI study.
626. Associations of gestational age and birth anthropometric indicators with brain white matter maturation in full-term neonates.
627. Test-retest reliability of Diffusion Tensor Imaging metrics in neonates.
628. Measurement of lateral ventricle volume of normal infant based on magnetic resonance imaging.
629. Very preterm children at risk for developmental coordination disorder have brain alterations in motor areas.
630. Arterial spin-labeling magnetic resonance imaging of brain maturation in early childhood: Mathematical model fitting to assess age-dependent change of cerebral blood flow.
631. Abnormal Microstructural Development of the Cerebral Cortex in Neonates With Congenital Heart Disease Is Associated With Impaired Cerebral Oxygen Delivery.
632. Gut microbiome and brain functional connectivity in infants-a preliminary study focusing on the amygdala.
633. Changes in brain perfusion in successive arterial spin labeling MRI scans in neonates with hypoxic-ischemic encephalopathy.
634. Network based statistics reveals trophic and neuroprotective effect of early high dose erythropoetin on brain connectivity in very preterm infants.
635. Fixel-based analysis of the preterm brain: Disentangling bundle-specific white matter microstructural and macrostructural changes in relation to clinical risk factors.
636. Brain microstructural development in neonates with critical congenital heart disease: An atlas-based diffusion tensor imaging study.
637. Early assessment of lateralization and sex influences on the microstructure of the white matter corticospinal tract in healthy term neonates.
638. Altered lateralization of dorsal language tracts in 6-week-old infants at risk for autism.
639. Age-dynamic networks and functional correlation for early white matter myelination.
640. White matter microstructural development and cognitive ability in the first 2 years of life.
641. Proper timing for the evaluation of neonatal brain white matter development: a diffusion tensor imaging study.
642. Alterations in Resting-State Networks Following In Utero Selective Serotonin Reuptake Inhibitor Exposure in the Neonatal Brain.
643. Mapping the asynchrony of cortical maturation in the infant brain: A MRI multi-parametric clustering approach.
644. Maternal Cortisol Concentrations During Pregnancy and Sex-Specific Associations With Neonatal Amygdala Connectivity and Emerging Internalizing Behaviors.
645. Longitudinal study of neonatal brain tissue volumes in preterm infants and their ability to predict neurodevelopmental outcome.
646. Different patterns of cortical maturation before and after 38 weeks gestational age demonstrated by diffusion MRI in vivo.
647. Maternal Interleukin-6 concentration during pregnancy is associated with variation in frontolimbic white matter and cognitive development in early life.
648. Music processing in preterm and full-term newborns: A psychophysiological interaction (PPI) approach in neonatal fMRI.
649. Neural correlates of gentle skin stroking in early infancy.
650. Plasma cholesterol levels and brain development in preterm newborns.
651. Nutritive sucking abnormalities and brain microstructural abnormalities in infants with established brain injury: a pilot study.
652. Cesarean Delivery Impacts Infant Brain Development.
653. Brain Development Measured With MRI in Children With Down Syndrome Correlates With Blood Biochemical Biomarkers.
654. Left temporal plane growth predicts language development in newborns with congenital heart disease.
655. Father-infant interactions and infant regional brain volumes: A cross-sectional MRI study.
656. Clinical Factors That Affect the Relationship between Head Circumference and Brain Volume in Very-Low-Birth-Weight Infants.
657. MR diffusion changes in the perimeter of the lateral ventricles demonstrate periventricular injury in post-hemorrhagic hydrocephalus of prematurity.
658. A PRELIMINARY VOLUMETRIC MRI STUDY OF AMYGDALA AND HIPPOCAMPAL SUBFIELDS IN AUTISM DURING INFANCY.
659. The dynamics of cortical folding waves and prematurity-related deviations revealed by spatial and spectral analysis of gyrification.
660. Impaired hippocampal development and outcomes in very preterm infants with perinatal brain injury.
661. Revealing Developmental Regionalization of Infant Cerebral Cortex Based on Multiple Cortical Properties.
662. Long-term Influences of Prenatal Maternal Depressive Symptoms on the Amygdala-Prefrontal Circuitry of the Offspring From Birth to Early Childhood.
663. Neonatal amygdalae and hippocampi are influenced by genotype and prenatal environment, and reflected in the neonatal DNA methylome.
664. Developmental changes in functional brain networks from birth through adolescence.
665. Polygenic risk for neuropsychiatric disease and vulnerability to abnormal deep grey matter development.
666. Early white matter development is abnormal in tuberous sclerosis complex patients who develop autism spectrum disorder.
667. A Longitudinal MRI Study of Amygdala and Hippocampal Subfields for Infants with Risk of Autism.
668. Quantitative MRI study of infant regional brain size following surgery for long-gap esophageal atresia requiring prolonged critical care.
669. Nutrition, Growth, Brain Volume, and Neurodevelopment in Very Preterm Children.
670. Prediction of Gait Impairment in Toddlers Born Preterm From Near-Term Brain Microstructure Assessed With DTI, Using Exhaustive Feature Selection and Cross-Validation.
671. Development and Emergence of Individual Variability in the Functional Connectivity Architecture of the Preterm Human Brain.
672. Early Pain Exposure Influences Functional Brain Connectivity in Very Preterm Neonates.
673. Postoperative brain volumes are associated with one-year neurodevelopmental outcome in children with severe congenital heart disease.
674. Developmental topography of cortical thickness during infancy.
675. Resting-State fMRI Networks in Children with Tuberous Sclerosis Complex.
676. Quantitative Analysis of Punctate White Matter Lesions in Neonates Using Quantitative Susceptibility Mapping and R2* Relaxation.
677. White matter development in infants at risk for schizophrenia.
678. Diffusion Tensor MRI of White Matter of Healthy Full-term Newborns: Relationship to Neurodevelopmental Outcomes.
679. What is the Functional Difference Between Sagittal With Metopic and Isolated Sagittal Craniosynotosis?
680. The relationship between biological and psychosocial risk factors and resting-state functional connectivity in 2-month-old Bangladeshi infants: A feasibility and pilot study.
681. Impact of Perioperative Brain Injury and Development on Feeding Modality in Infants With Single Ventricle Heart Disease.
682. Early Detection of Cerebral Palsy Using Sensorimotor Tract Biomarkers in Very Preterm Infants.
683. Hub distribution of the brain functional networks of newborns prenatally exposed to maternal depression and SSRI antidepressants.
684. Isolated periventricular pseudocysts do not affect white matter microstructure development in neonatal stage: A retrospective case-control diffusion tensor imaging study.
685. Functional and structural connectivity of the brain in very preterm babies: relationship with gestational age and body and brain growth.
686. Social Brain Functional Maturation in Newborn Infants With and Without a Family History of Autism Spectrum Disorder.
687. Resting-state network complexity and magnitude changes in neonates with severe hypoxic ischemic encephalopathy.
688. Structural Magnetic Resonance Imaging-Based Brain Morphology Study in Infants and Toddlers With Down Syndrome: The Effect of Comorbidities.
689. System-Specific Patterns of Thalamocortical Connectivity in Early Brain Development as Revealed by Structural and Functional MRI.
690. Cerebellar Functional Connectivity in Term- and Very Preterm-Born Infants.
691. Environmental Influences on Infant Cortical Thickness and Surface Area.
692. Familial risk of autism alters subcortical and cerebellar brain anatomy in infants and predicts the emergence of repetitive behaviors in early childhood.
693. Differential cortical microstructural maturation in the preterm human brain with diffusion kurtosis and tensor imaging.
694. Brain functional development separates into three distinct time periods in the first two years of life.
695. Bronchopulmonary Dysplasia Is Associated with Altered Brain Volumes and White Matter Microstructure in Preterm Infants.
696. Early extra-uterine exposure alters regional cerebellar growth in infants born preterm.
697. Newborn amygdala connectivity and early emerging fear.
698. Characterisation of brain volume and microstructure at term-equivalent age in infants born across the gestational age spectrum.
699. Brain structure and neurological and behavioural functioning in infants born preterm.
700. White matter injury predicts disrupted functional connectivity and microstructure in very preterm born neonates.
701. Associations of age and sex with brain volumes and asymmetry in 2-5-week-old infants.
702. Accelerated Small-World Property of Structural Brain Networks in Preterm Infants at Term-Equivalent Age.
703. Quantitative tract-based white matter heritability in 1- and 2-year-old twins.
704. The Role of Diffusion Tensor Imaging in Detecting Hippocampal Injury Following Neonatal Hypoxic-Ischemic Encephalopathy.
705. First-year development of modules and hubs in infant brain functional networks.
706. Neonate and infant brain development from birth to 2 years assessed using MRI-based quantitative susceptibility mapping.
707. Restricted and Repetitive Behavior and Brain Functional Connectivity in Infants at Risk for Developing Autism Spectrum Disorder.
708. Sex-specific alterations in preterm brain.
709. Early breast milk exposure modifies brain connectivity in preterm infants.
710. Development of Amygdala Functional Connectivity During Infancy and Its Relationship With 4-Year Behavioral Outcomes.
711. Changes in neonatal regional brain volume associated with preterm birth and perinatal factors.
712. Age-specific gray and white matter DTI atlas for human brain at 33, 36 and 39 postmenstrual weeks.
713. Structural network maturation of the preterm human brain.
714. White matter injury in term neonates with congenital heart diseases: Topology & comparison with preterm newborns.
715. Early life predictors of brain development at term-equivalent age in infants born across the gestational age spectrum.
716. Amygdala Functional Connectivity and Negative Reactive Temperament at Age 4 Months.
717. Lack of neural evidence for implicit language learning in 9-month-old infants at high risk for autism.
718. Diffusion-MRI-based regional cortical microstructure at birth for predicting neurodevelopmental outcomes of 2-year-olds.
719. Delayed maturation of the structural brain connectome in neonates with congenital heart disease.
720. Maternal cortisol is associated with neonatal amygdala microstructure and connectivity in a sexually dimorphic manner.
721. Cortical morphology at birth reflects spatiotemporal patterns of gene expression in the fetal human brain.
722. DTI parameters in neonates with hypoxic-ischemic encephalopathy after total body hypothermia.
723. Prenatal opioid exposure is associated with smaller brain volumes in multiple regions.
724. Partial Support for an Interaction Between a Polygenic Risk Score for Major Depressive Disorder and Prenatal Maternal Depressive Symptoms on Infant Right Amygdalar Volumes.
725. Disruption and Compensation of Sulcation-based Covariance Networks in Neonatal Brain Growth after Perinatal Injury.
726. Association of Cumulative Paternal Early Life Stress With White Matter Maturation in Newborns.
727. Prenatal stress exposure and multimodal assessment of amygdala-medial prefrontal cortex connectivity in infants.
728. Brain Development in Infants of Mothers With Gestational Diabetes Mellitus: A Diffusion Tensor Imaging Study.
729. Reduced fractional anisotropy in projection, association, and commissural fiber networks in neonates with prenatal methamphetamine exposure.
730. Neonatal genetic epilepsies display convergent white matter microstructural abnormalities.
731. Innate connectivity patterns drive the development of the visual word form area.
732. Association of early skin breaks and neonatal thalamic maturation: A modifiable risk?
733. Adults vs. neonates: Differentiation of functional connectivity between the basolateral amygdala and occipitotemporal cortex.
734. Neurocranium thickness mapping in early childhood.
735. A Novel Method for High-Dimensional Anatomical Mapping of Extra-Axial Cerebrospinal Fluid: Application to the Infant Brain.
736. Location and Size of Preterm Cerebellar Hemorrhage and Childhood Development.
737. Neural Correlates of Voice Perception in Newborns and the Influence of Preterm Birth.
738. Novel diffuse white matter abnormality biomarker at term-equivalent age enhances prediction of long-term motor development in very preterm children.
739. Cerebral Pulsed Arterial Spin Labeling Perfusion Weighted Imaging Predicts Language and Motor Outcomes in Neonatal Hypoxic-Ischemic Encephalopathy.
740. Early Prediction of Cognitive Deficit in Very Preterm Infants Using Brain Structural Connectome With Transfer Learning Enhanced Deep Convolutional Neural Networks.
741. Breastfeeding improves dynamic reorganization of functional connectivity in preterm infants: a temporal brain network study.
742. Interleukin-8 dysregulation is implicated in brain dysmaturation following preterm birth.
743. Investigating altered brain development in infants with congenital heart disease using tensor-based morphometry.
744. Maternal Anxiety and Depression during Late Pregnancy and Newborn Brain White Matter Development.
745. Sex-specific association between infant caudate volumes and a polygenic risk score for major depressive disorder.
746. Head Ultrasound Resistive Indices Are Associated With Brain Injury on Diffusion Tensor Imaging Magnetic Resonance Imaging in Neonates With Hypoxic-Ischemic Encephalopathy.
747. The emergence of a functionally flexible brain during early infancy.
748. The developing Human Connectome Project (dHCP) automated resting-state functional processing framework for newborn infants.
749. Inferring pain experience in infants using quantitative whole-brain functional MRI signatures: a cross-sectional, observational study.
750. Non-negative data-driven mapping of structural connections with application to the neonatal brain.
751. Developmental score of the infant brain: characterizing diffusion MRI in term- and preterm-born infants.
752. Longitudinal growth of the basal ganglia and thalamus in very preterm children.
753. Neonatal brain abnormalities and brain volumes associated with goal setting outcomes in very preterm 13-year-olds.
754. Newborn white matter microstructure moderates the association between maternal postpartum depressive symptoms and infant negative reactivity.
755. Changes of Dynamic Functional Connectivity Associated With Maturity in Late Preterm Infants.
756. Feed-forward neural networks using cerebral MR spectroscopy and DTI might predict neurodevelopmental outcome in preterm neonates.
757. Early application of caffeine improves white matter development in very preterm infants.
758. Neonatal brain volume as a marker of differential susceptibility to parenting quality and its association with neurodevelopment across early childhood.
759. Non-right-handedness in children born extremely preterm: Relation to early neuroimaging and long-term neurodevelopment.
760. Neonatal hippocampal volume moderates the effects of early postnatal enrichment on cognitive development.
761. Early microstructure of white matter associated with infant attention.
762. Ventricular and total brain volumes in infants with congenital heart disease: a longitudinal study.
763. Functional brain connectivity in ex utero premature infants compared to in utero fetuses.
764. Neural Transcription Correlates of Multimodal Cortical Phenotypes during Development.
765. Limbic white matter structural integrity at 3 months prospectively predicts negative emotionality in 9-month-old infants: a preliminary study.
766. Increase in Brain Volumes after Implementation of a Nutrition Regimen in Infants Born Extremely Preterm.
767. Infant Corpus Callosum Size After Surgery and Critical Care for Long-Gap Esophageal Atresia: Qualitative and Quantitative MRI.
768. Altered structural brain networks at term-equivalent age in preterm infants with grade 1 intraventricular hemorrhage.
769. Morphine affects brain activity and volumes in preterms: An observational multi-center study.
770. Sex differences associated with corpus callosum development in human infants: A longitudinal multimodal imaging study.
771. Peak Width of Skeletonized Water Diffusion MRI in the Neonatal Brain.
772. Objectively Diagnosed Diffuse White Matter Abnormality at Term Is an Independent Predictor of Cognitive and Language Outcomes in Infants Born Very Preterm.
773. Regional brain volumes, microstructure and neurodevelopment in moderate-late preterm children.
774. Connectivity at the origins of domain specificity in the cortical face and place networks.
775. Texture analysis of deep medullary veins on susceptibility-weighted imaging in infants: evaluating developmental and ischemic changes.
776. Antecedents of Objectively Diagnosed Diffuse White Matter Abnormality in Very Preterm Infants.
777. Tracking regional brain growth up to age 13 in children born term and very preterm.
778. Anatomo-functional correlates of auditory development in infancy.
779. Automated brain morphometric biomarkers from MRI at term predict motor development in very preterm infants.
780. Neonatal white matter tract microstructure and 2-year language outcomes after preterm birth.
781. More than meets the eye: Longitudinal visual system neurodevelopment in very preterm children and anophthalmia.
782. Creative music therapy to promote brain function and brain structure in preterm infants: A randomized controlled pilot study.
783. Early alterations in cortical and cerebellar regional brain growth in Down Syndrome: An in vivo fetal and neonatal MRI assessment.
784. Myelin water imaging and R(2) (*) mapping in neonates: Investigating R(2) (*) dependence on myelin and fibre orientation in whole brain white matter.
785. Music enhances structural maturation of emotional processing neural pathways in very preterm infants.
786. Early prediction of unilateral cerebral palsy in infants at risk: MRI versus the hand assessment for infants.
787. Two-dimensional ultrasound measurements vs. magnetic resonance imaging-derived ventricular volume of preterm infants with germinal matrix intraventricular haemorrhage.
788. Therapeutic hypothermia for neonatal hypoxic-ischaemic encephalopathy in India (THIN study): a randomised controlled trial.
789. Neonatal Functional and Structural Connectivity Are Associated with Cerebral Palsy at Two Years of Age.
790. Feasibility of oscillating and pulsed gradient diffusion MRI to assess neonatal hypoxia-ischemia on clinical systems.
791. Microstructure of the Dorsal Anterior Cingulum Bundle in Very Preterm Neonates Predicts the Preterm Behavioral Phenotype at 5 Years of Age.
792. Structural and functional brain network alterations in prenatal alcohol exposed neonates.
793. Shedding light on excessive crying in babies.
794. Development of Dynamic Functional Architecture during Early Infancy.
795. Development of Microstructural and Morphological Cortical Profiles in the Neonatal Brain.
796. Associations of Maternal Prenatal Drug Abuse With Measures of Newborn Brain Structure, Tissue Organization, and Metabolite Concentrations.
797. Early parenting is associated with the developing brains of children born very preterm.
798. Exploring functional brain activity in neonates: A resting-state fMRI study.
799. Newborn left amygdala volume associates with attention disengagement from fearful faces at eight months.
800. Mental development is associated with cortical connectivity of the ventral and nonspecific thalamus of preterm newborns.
801. Functional dissection of prenatal drug effects on baby brain and behavioral development.
802. Regional impairment of cortical and deep gray matter perfusion in preterm neonates with low-grade germinal matrix-intraventricular hemorrhage: an ASL study.
803. Heterogeneity in Brain Microstructural Development Following Preterm Birth.
804. Brain microstructure and morphology of very preterm-born infants at term equivalent age: Associations with motor and cognitive outcomes at 1 and 2 years.
805. Brain connectivity and socioeconomic status at birth and externalizing symptoms at age 2 years.
806. Emerging atypicalities in functional connectivity of language-related networks in young infants at high familial risk for ASD.
807. Individual Variation of Human Cortical Structure Is Established in the First Year of Life.
808. Mechanical Ventilation Duration, Brainstem Development, and Neurodevelopment in Children Born Preterm: A Prospective Cohort Study.
809. Brain tissue volumes at term-equivalent age are associated with early motor behavior in very preterm infants.
810. Sleep Onset Problems and Subcortical Development in Infants Later Diagnosed With Autism Spectrum Disorder.
811. Emerging functional connectivity differences in newborn infants vulnerable to autism spectrum disorders.
812. Maternal Dietary Intake of Omega-3 Fatty Acids Correlates Positively with Regional Brain Volumes in 1-Month-Old Term Infants.
813. Predicting motor outcome in preterm infants from very early brain diffusion MRI using a deep learning convolutional neural network (CNN) model.
814. Extra-axial Cerebrospinal Fluid Relationships to Infant Brain Structure, Cognitive Development, and Risk for Schizophrenia.
815. Preterm infants with isolated cerebellar hemorrhage show bilateral cortical alterations at term equivalent age.
816. Altered Brain Structure in Infants with Turner Syndrome.
817. Altered local cerebellar and brainstem development in preterm infants.
818. Neonatal Brain Microstructure and Machine-Learning-Based Prediction of Early Language Development in Children Born Very Preterm.
819. Resting-state networks of the neonate brain identified using independent component analysis.
820. White matter extension of the Melbourne Children's Regional Infant Brain atlas: M-CRIB-WM.
821. Improved brain growth and microstructural development in breast milk-fed very low birth weight premature infants.
822. Functional connectivity correlates of infant and early childhood cognitive development.
823. Is Low-Grade Intraventricular Hemorrhage in Very Preterm Infants an Innocent Condition? Structural and Functional Evaluation of the Brain Reveals Regional Neurodevelopmental Abnormalities.
824. Relating anthropometric indicators to brain structure in 2-month-old Bangladeshi infants growing up in poverty: A pilot study.
825. Individual identification and individual variability analysis based on cortical folding features in developing infant singletons and twins.
826. Multiscale Structure-Function Gradients in the Neonatal Connectome.
827. Parental age effects on neonatal white matter development.
828. Perinatal thalamic injury: MRI predictors of electrical status epilepticus in sleep and long-term neurodevelopment.
829. Neonatal morphometric similarity mapping for predicting brain age and characterizing neuroanatomic variation associated with preterm birth.
830. Variable functional connectivity architecture of the preterm human brain: Impact of developmental cortical expansion and maturation.
831. Correlation of lateral ventricular size and deep gray matter volume in MRI at term equivalent age with neurodevelopmental outcome at a corrected age of 24 months and with handedness in preterm infants.
832. Early cortical maturation predicts neurodevelopment in very preterm infants.
833. Maternal Prenatal Stress Is Associated With Altered Uncinate Fasciculus Microstructure in Premature Neonates.
834. Prenatal socioeconomic status and social support are associated with neonatal brain morphology, toddler language and psychiatric symptoms.
